# Nanomaterial-Assisted Physical Mass Loading and Signal Amplification Strategies for Exosome Isolation and Sensing in Liquid Biopsy: A Review

**DOI:** 10.3390/bios16070384

**Published:** 2026-07-14

**Authors:** Sumedha Nitin Prabhu

**Affiliations:** Biomedical Science & Engineering Programme, School of Medicine, The Chinese University of Hong Kong, Shenzhen 518172, China; sumedhanitinprabhu@cuhk.edu.cn

**Keywords:** exosomes, extracellular vesicles, liquid biopsy, nanomaterial-assisted sensing, physical mass loading, non-gravimetric signal amplification, exosome isolation, biosensors, clinical translation, matrix effects

## Abstract

Exosomes and small extracellular vesicles are promising liquid-biopsy biomarkers because they carry molecular information from their cells of origin and can be accessed from minimally invasive biofluids. Reliable separation and detection are made more difficult by their small size, low abundance, diverse composition, and co-occurrence with lipoproteins, protein aggregates, and other extracellular particles. To improve exosome enrichment, capture, and sensing, nanomaterial-assisted techniques have become crucial. Using a mechanism-based approach that differentiates between non-gravimetric signal amplification and genuine physical mass loading, this study offers an organized comparison of nanomaterial-enabled exosome sensing techniques. This distinction is helpful because different transducers measure different physical quantities: while optical, electrochemical, fluorescent, catalytic, and nucleic acid-based platforms typically benefit from enhanced signal generation rather than increased mass, resonant and gravimetric sensors benefit from increased inertial or surface-bound mass. In terms of amplification mechanism, transducer compatibility, sample-matrix tolerance, workflow complexity, and translational maturity, the review contrasts metallic nanoparticles, magnetic systems, metal–organic frameworks, carbon and two-dimensional materials, quantum dots, upconversion nanomaterials, DNA nanostructures, and polymer-based platforms. The gap between analytical sensitivity and clinical utility, including separation purity, recovery, biological heterogeneity, pre-analytical variability, interference from complex biofluids, and the need for uniform validation, is given special focus. The review concludes that no single nanomaterial or amplification method is universally optimal; instead, platform-aware, application-specific integration of isolation, amplification, and validation techniques is necessary for clinically meaningful exosome sensing.

## 1. Introduction

The goal of biosensing technology is to accurately translate molecular recognition events into quantifiable physical signals [1]. When the target analytes, such as exosomes, are present at extremely low concentrations, have nanoscale dimensions, and are embedded in intricate biological matrices containing interfering species, the transduction problem in liquid biopsy becomes especially acute [2]. Exosomes are usually between 30 and 150 [3] nm in diameter, have low intrinsic mass, and have diverse surface compositions that significantly overlap with lipoproteins, protein aggregates, and non-vesicular nanoparticles. Consequently, poor signal-to-noise ratios [4,5], diffusion-limited binding kinetics, and inadequate analytical sensitivity for early-stage disease diagnosis [6] are common limitations of direct exosome detection using traditional biosensors [7].

Due to their wide application and long history of usage, conventional exosome extraction techniques, particularly differential ultracentrifugation, have been fundamental to extracellular vesicle research. Nevertheless, these procedures are not ideal for routine translational or point-of-care deployment, even if they are widely used. Long processing periods, specialized equipment, large sample volumes, multiple centrifugation stages that may cause vesicle loss, co-isolation of protein aggregates and lipoproteins, and operator-dependent variability are all common requirements of ultracentrifugation. Because of this, ultracentrifugation should not be considered a universally ideal benchmark for all exosome applications, even though it is frequently regarded as a typical reference method in the literature. Alternative nanomaterial-assisted systems that combine enrichment, purification, and signal amplification in faster, more scalable, and perhaps higher-recovery procedures have been developed to address this constraint.

A transducer is an intermediate whose output signal scales with a physical perturbation brought on by target binding. It works by sensing variations in mass, refractive index, electrical impedance, catalytic activity, or optical emission, which is at the heart of most biosensing platforms. However, even sophisticated transduction modalities often fail to detect the absolute disruption at the sensor interface caused by nanoscale vesicles such as exosomes. Mass magnification techniques, which intentionally increase the effective physical footprint of a single molecular recognition event by integrating nanomaterials [8], have emerged in response to this fundamental restriction [9,10].

To transform weak biorecognition signals into macroscopically resolvable outputs, nanostructure-assisted mass magnification leverages the unique physicochemical characteristics of nanoscale materials, such as high density, large surface-to-volume ratio, plasmonic coupling, catalytic activity, magnetic susceptibility, and quantum confinement. Crucially, by linking biological binding events to nonlinear amplification mechanisms such as enzymatic cascades, catalytic turnover, collective nanoparticle aggregation, or enhanced electromagnetic-field confinement, this strategy radically alters the transduction landscape rather than merely boosting signal intensity [11].

Nanomaterial-assisted amplification in exosome biosensing can occur through at least two distinct routes: (i) true physical mass loading, which is directly relevant to mass-sensitive resonant platforms; and (ii) non-gravimetric signal amplification, including plasmonic, catalytic, electrochemical, and nucleic-acid-mediated enhancement mechanisms. First, each trapped vesicle’s apparent mass, optical cross-section, or electrochemical activity is significantly increased by nanomaterials acting as secondary or tertiary labels. Second, they serve as active transduction enhancers, facilitating multiplexed readouts across the optical, electrical, magnetic, and mechanical domains while accelerating binding kinetics and overcoming diffusion barriers. To achieve ultrasensitive, rapid, and clinically feasible exosome detection, not only theoretical calculations [12], but a nanomaterial-enabled physical mass amplification has emerged as a key design element [13].

With an emphasis on how material composition, morphology, and surface chemistry directly affect signal amplification efficiency, biocompatibility, and translational potential, this study methodically investigates nanostructure-based mass magnification techniques for exosome isolation and sensing.

**Figure 1:**
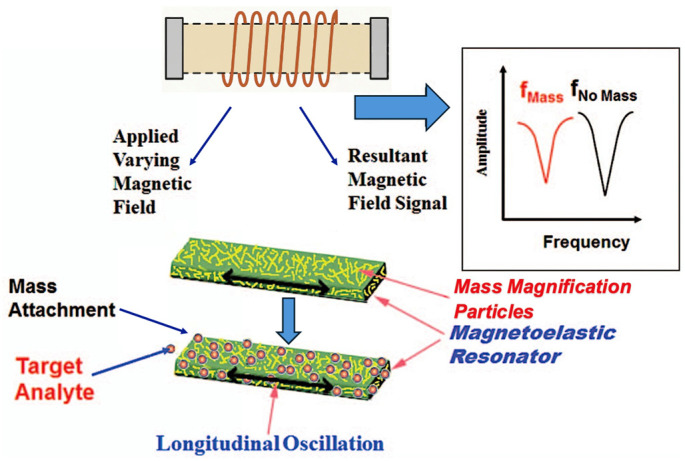
Principle of operation of magnetoelastic biosensors.

The original motivation for this review arose from the challenge of detecting exosomes with mass-sensitive resonant biosensors, particularly MEMS-based magnetoelastic platforms, in which the intrinsic mass of individual exosomes is often insufficient to generate readily resolvable resonance-frequency shifts. In that specific context, physical mass loading through dense secondary nanomaterials is of primary importance. Figure 1 presents the principle of operation of magnetoelastic biosensors. However, during the preparation of this review, it became clear that the broader exosome literature also includes multiple non-gravimetric amplification strategies—such as plasmonic, catalytic, chemiluminescent, electrochemical, and nucleic-acid-mediated amplification—that, although not strictly “mass amplification” in the physical sense, can still enhance detectability and are often coupled to capture or enrichment steps. Accordingly, this review explicitly distinguishes between true physical mass amplification and broader signal-amplification strategies, while retaining a particular emphasis on their relevance to mass-limited exosome analysis.

In this review, the term “physical mass amplification” refers to strategies that increase the effective gravimetric or inertial load associated with exosome capture, which is particularly important for mass-sensitive resonant transducers such as magnetoelastic, acoustic, and microcantilever sensors. By contrast, plasmonic, catalytic, chemiluminescent, electrochemical, and nucleic-acid-mediated approaches are treated here as signal-amplification or transduction-enhancement strategies rather than true mass amplification. This distinction is important both conceptually and translationally because different amplification routes impose different design constraints, material requirements, and sensor compatibilities.

In this review, the term “exosome” is used when referring to studies that used this terminology, while “EV” or “sEV” is retained when used by the original authors or when the broader vesicle population is intended.

Accordingly, the purpose of this review is not merely to catalog exosome detection methods but to provide a material-guided framework for selecting amplification strategies, especially physical mass-loading approaches, for applications in mass-sensitive biosensing, while also considering complementary non-gravimetric signal-amplification mechanisms that may improve analytical performance in integrated platforms.

## 2. Review Methodology and Literature Selection Strategy

This review was conducted as a structured review with systematic search elements rather than as a formal PRISMA-registered systematic review or meta-analysis. The purpose was to identify representative and mechanistically relevant studies describing nanomaterial-assisted exosome or extracellular vesicle isolation, enrichment, detection, and signal amplification, with particular attention to platforms relevant to physical mass loading and mass-sensitive biosensing.

Web of Science, Scopus, PubMed, and Google Scholar were used to conduct literature searches. Publications up to 2026 were included in the search. Exosome, extracellular vesicle, small extracellular vesicle, liquid biopsy, nanomaterial, nanoparticle, mass amplification, mass loading, signal amplification, biosensor, magnetoelastic sensor, acoustic sensor, QCM, SAW, microcantilever, electrochemical, plasmonic, fluorescence, chemiluminescence, metal–organic framework, MOF, MXene, graphene, carbon nanotube, quantum dot, upconversion nanoparticle, DNA walker, rolling circle amplification, and polymer nanostructure were among the primary search terms.

Studies that satisfied at least one of the following requirements were included:

(i) the study employed nanomaterials for exosome/EV isolation, enrichment, capture, or purification;
(ii) the study employed nanomaterials as labels, transduction enhancers, reporter carriers, or signal-amplifying components for exosome/EV detection;
(iii) the study offered analytical performance metrics like dynamic range, limit of detection, sample matrix, recovery, or real-sample validation; or
(iv) the study offered mechanistic insight pertinent to physical mass loading, mass-sensitive detection, or non-gravimetric signal amplification.

Studies that solely addressed non-exosomal biomarkers, general therapeutic delivery, EV biogenesis, tissue repair, or intracellular uptake without a clear connection to exosome/EV isolation, enrichment, or sensing were not included in the primary comparative tables. When these studies provided essential mechanistic background, they were either omitted or cited only briefly. Detection limits were not regarded as directly comparable performance rankings, since assay designs, targets, sample matrices, and reporting protocols vary significantly across the literature. Rather, they were interpreted according to the transducer mechanism, the nanomaterial’s capability to amplify exosome mass, the assay methodology, the sample type, and the target description.

## 3. Nanomaterial-Assisted Amplification Strategies for Exosome Isolation and Sensing

As exosomes are microscopic, compositionally variable, and often present at low abundance in complex biological fluids, their characterization poses a fundamental analytical challenge. Because of this, there are weak interfacial contacts, low local analyte density, nonspecific background, and, in the case of resonant or gravimetric transducers, insufficient intrinsic mass to provide a reliable quantifiable response sometimes impede direct detection. As a result, nanomaterials have evolved from passive labels to active amplification components that can enhance signal transduction, exosome capture and enrichment, interfacial transport, and, in some cases, physical mass loading.

It is crucial to distinguish between non-gravimetric signal amplification, in which nanomaterials improve detectability through plasmonic, catalytic, chemiluminescent, fluorescent, electrochemical, or nucleic acid-mediated mechanisms, and physical mass amplification, which directly increases the gravimetric or inertial load relevant to mass-sensitive platforms. For researchers creating resonant sensing platforms, such as magnetoelastic, acoustic, and cantilever-based sensors, where exosome mass alone might not be adequate for accurate readout, this distinction is particularly crucial. However, because they enhance exosome detectability in integrated processes and may supplement physical enrichment or capture, many non-gravimetric amplification techniques remain extremely important.

Building on the distinction introduced above, nanomaterial-assisted exosome analysis can be organized into physical mass-loading strategies and non-gravimetric signal-amplification strategies. The following sections compare these approaches according to amplification mechanism, material class, transducer compatibility, and translational feasibility.

In biosensing, mass magnification is a range of amplification mechanisms arising from multiple interactions between nanomaterials and biomolecules rather than a single event. Magnification can result from enhanced electromagnetic coupling (e.g., plasmonic or surface plasmon resonance (SPR) sensors), increased inertial mass loading (e.g., acoustic or mechanical sensors), catalytic signal multiplication (e.g., enzyme-mimicking nanozymes), or collective optical and electronic effects (e.g., nanoparticle aggregation, quantum emission), depending on the transduction modality.

**Figure 2:**
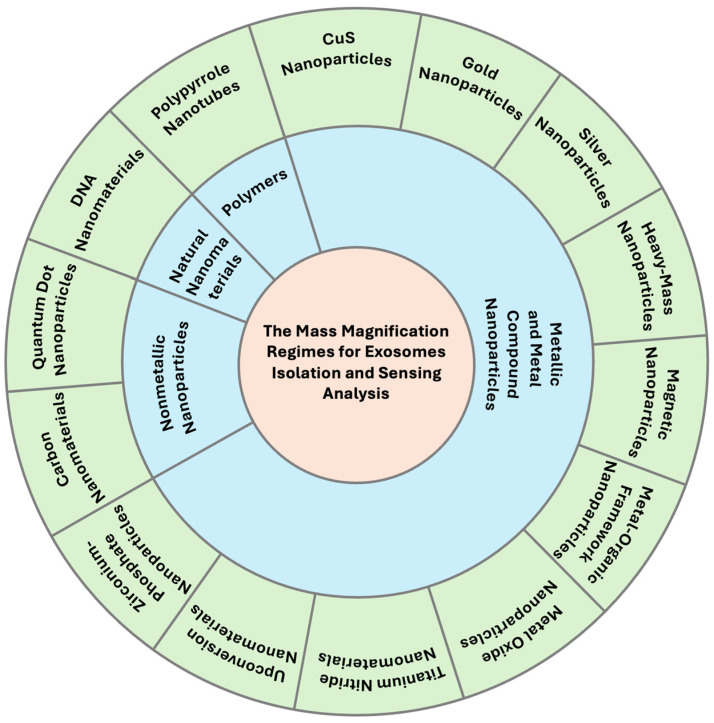
The mass magnification regimes for exosome isolation and sensing analysis.

Due to their physical dimensions matching those of biological entities such as exosomes, nanostructures are particularly well-suited for these activities, enabling close interfacial contact without steric exclusion. Additionally, their surfaces can be precisely designed [14] with peptides [15], nucleic acids [16], aptamers [17], or antibodies [18] to guarantee specific recognition while also permitting downstream signal amplification. Figure 2 describes the mass magnification regimes for exosome isolation and sensing analysis.

### 3.1. Exosome Isolation Challenges Relevant to Nanomaterial-Assisted Sensing

Reliable exosome detection depends greatly on the quality of upstream isolation and enrichment. In clinical biofluids, exosomes coexist with lipoproteins, protein aggregates, ribonucleoprotein complexes, apoptotic bodies, microvesicles, and other extracellular nanoparticles that share overlapping size, density, and surface features. As a result, great analytical sensitivity at the sensor level does not necessarily translate into precise quantification of disease-associated vesicles. Incomplete recovery, co-isolation of contaminants, marker heterogeneity, variable antibody or aptamer capture efficiency, nonspecific adsorption, and variations arising from sample collection, anticoagulant selection, storage, freeze–thaw cycles, centrifugation, filtration, and pre-clearing procedures are among the main isolation-related difficulties.

Although it is still commonly employed, conventional ultracentrifugation is laborious, operator-dependent, and prone to co-isolation of non-vesicular species and vesicle loss. There are benefits to size-exclusion chromatography, precipitation, immunoaffinity capture, microfluidics, and nanomaterial-assisted enrichment, but there are also method-specific biases. Affinity-based methods, for instance, can increase specificity but can only enrich marker-positive subpopulations, whereas charge- or size-based methods might offer greater recovery but less molecular specificity. As a result, translational exosome biosensing necessitates reporting recovery, purity, vesicle characterization, matrix effects, and repeatability across clinically relevant sample types in addition to the limit of detection.

In this regard, nanomaterials should be assessed not only as signal amplifiers but also as elements that affect background suppression, isolation quality, and workflow compatibility. Platforms that combine enrichment, interference reduction, and signal readout while maintaining vesicle integrity and delivering consistent performance in real biofluids are likely the most therapeutically beneficial.

**I. Metallic and Metal Compound Nanoparticles**: Considering their strong interaction with electromagnetic fields, tunable electronic structure, and high atomic density, metallic and metal compound nanoparticles are the most studied class of mass magnification agents. Metals offer unmatched benefits for sensing: they enable magnetic or electrochemical manipulation, promote localized surface plasmon resonances, accelerate redox reactions, and significantly increase effective mass loading.

Metallic nanoparticles often serve as signal-transduction bridges in exosome biosensing, converting nanoscale binding events into amplified optical, electrical, or catalytic outputs. They are especially useful for mass-sensitive platforms such as surface acoustic wave (SAW), quartz crystal microbalance (QCM), and plasmonic sensors because of their high density relative to biomolecules. High concentrations of recognition ligands can be present simultaneously on their surfaces, enabling multivalent binding that enhances selectivity and stabilizes vesicle capture.

By adding enzyme-mimetic activity, improved chemical stability, or multifunctional sensing modalities, metal compounds, such as metal sulfides, oxides, nitrides, and hybrid composites, further expand these capacities. When appropriately integrated, metallic nanostructures collectively represent the highest-mass and highest-impact amplification elements, frequently enabling single-exosome-level detection.

The material composition and functional mechanism of nanostructure-based mass magnification techniques are categorized in this area. Sensitivity limits, scalability, and compliance with clinical procedures are determined by fundamentally different amplification physics, which are reflected in this classification in addition to chemical variations.

(i) CuS Nanoparticles: Due to their strong chemiluminescence–catalytic coupling and high atomic density, which effectively convert exosome binding events into amplified photon output, copper sulfide nanoparticles are very useful for mass magnification. CuS-based systems can contribute both dense nanomaterial loading and strong catalytic/chemiluminescent signal amplification. Still, the dominant mechanism in many reported platforms is reporter amplification rather than gravimetric mass loading. They are logistically ideal for sensitive detection in complex biofluids, as their incorporation into microgels further boosts effective mass loading while preserving permeability.

**Figure 3:**
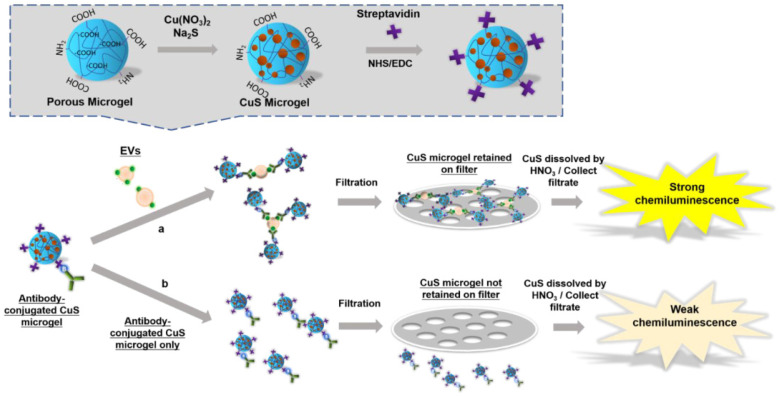
Schematic Illustration of CuS-MG Synthesis (**Upper Gray Panel**) and the CuS-MG-Based Assay for EV Quantification (**Lower Panel**) [19].

Extracellular vesicles (EVs) are cell-derived membranous vesicles found in almost all bodily fluids, including blood and urine, and transport a wide range of cargo molecules such as proteins, nucleic acids, and lipids. The significance of EVs in cell–cell communication and pathological processes is unclear, necessitating the development of sensitive, precise, and quick techniques for detecting and quantifying them in biological materials. Jiang et al. developed CuS-enclosed microgels that concentrate EVs carrying specific protein markers from complex biomatrices and produce strong chemiluminescence (CL), enabling sensitive detection of target EVs. These microgels successfully detected 10^4^ EV particles/mL by targeting EV proteins such as CD63 and human epidermal growth factor receptor 2 (HER2), with a dynamic range of up to 10^8^ particles/mL. The direct detection of EVs in human serum and cell culture media requires less sample preparation than the enzyme-linked immunosorbent assay (ELISA) and Western blot. The researchers’ approach can quickly quantify EVs in biological samples, facilitating disease monitoring and functional studies [19]. Figure 3 presents a schematic illustration of Cus-MG synthesis (upper gray panel) and the Cus-MG-based assay for EV quantification (lower panel).

To detect CD91 and CD151 on the surface of NSCLC exosomes with high sensitivity, An et al. developed a novel dual-fluorescence, dual-target biosensor based on copper nanoclusters (CuNCs) and hollow mesoporous silicon nanospheres (HMSNs). By loading Ce^3+^ and Fe^3+^ and conjugating a pH-responsive chitosan coating, HMSNs specifically control the fluorescence signals of red and blue glutathione-CuNCs. Ce^3+^ greatly increases the fluorescence signal of red CuNCs by causing their aggregation, while Fe^3+^ quenches the fluorescence signal of blue CuNCs through oxidation. A CD63 aptamer coupled to streptavidin magnetic beads is used to collect exosomes. The exosome surface proteins CD151 and CD91 are then targeted by HMSN-Ce^3+^-CS-antiCD151 and HMSN-Fe^3+^-CS-antiCD91 probes, respectively, enabling simultaneous detection of dual-fluorescence and dual-targIkkets. The authors then monitored and evaluated the change ratio of fluorescence signals, optimized the detection settings, and assessed the system’s sensitivity, stability, and linear range. Red and blue fluorescence systems had detection limits of 1058 particles/mL and 239 particles/mL, with linearity ranges of 4 × 10^3^–4 × 10^8^ and 7 × 10^2^–7 × 10^6^ particles/mL, respectively. Overall, this work presents a unique, highly sensitive, and less invasive method for early detection of NSCLC. The dual-fluorescence system’s complementary architecture expands its detection range and has translational potential [20].

(ii) Gold Nanoparticles: Gold nanoparticles’ high electron density, tunable plasmonic properties, and ease of surface functionalization with aptamers or antibodies make them an exceptionally stable and biocompatible platform for physical mass amplification. Exosome recognition events can be converted into macroscopically resolvable signals with less equipment because they can undergo aggregation-induced optical and electrochemical alterations.

**Figure 4:**
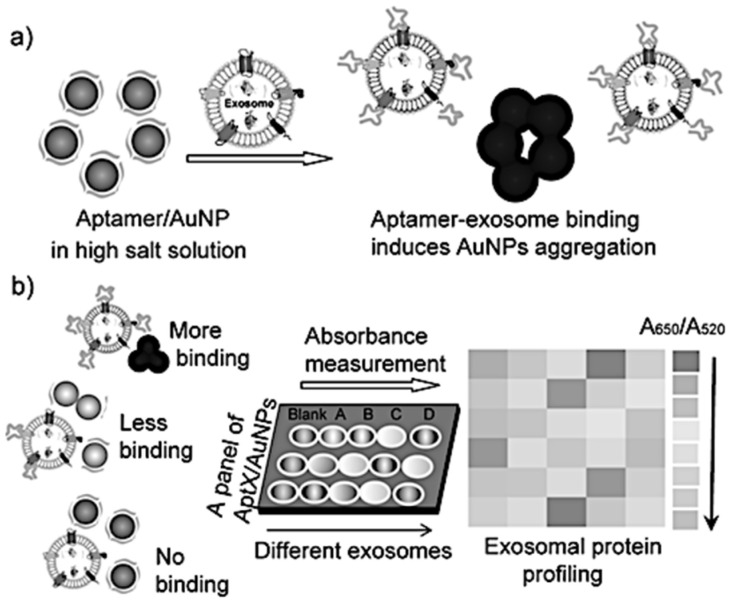
Working principle of the aptamer/AuNP complex for molecular profiling of exosomal proteins. (**a**) Schematic of the displacement of aptamers from gold nanoparticles by binding with exosome surface protein and the concomitant aggregation of gold nanoparticles. (**b**) Profiling of different exosome surface proteins with the aptamer/AuNP complex [21].

Exosomes are an emerging biomarker for cancer detection because they contain proteins that reflect the origins of the parent cells. Examining exosome surface proteins is an effective way to uncover a combination of biomarkers for cancer detection. Jiang et al. provided a sensor technology that can characterize exosome surface proteins in minutes using the naked eye. The sensor comprises a gold nanoparticle (AuNP) complexed with a panel of aptamers. The complexation of aptamers with AuNPs prevents aggregation of the nanoparticles in high-salt solution. Exosomes disrupt the non-specific, weaker binding between aptamers and AuNP. In contrast, the stronger, specific binding between the exosome surface protein and the aptamer displaces the aptamer from the AuNP surface, leading to AuNP aggregation. The aggregation of exosomes results in color changes and patterns that can be used to identify numerous proteins on their surfaces [21]. Figure 4 depicts the working principle of the aptamer/AuNP complex for molecular profiling of exosomal proteins.

More recently, Della Ventura et al. reported a plasmonic anti-aggregation strategy for exosome sensing using gold nanoparticles in a clinically oriented liquid-biopsy context. In contrast to aggregation-based colorimetric assays in which target binding promotes nanoparticle clustering, anti-aggregation formats rely on target-induced stabilization or inhibition of salt-induced aggregation, producing a measurable plasmonic color change. This strategy is attractive for translational exosome analysis because it can provide rapid optical readout, minimal instrumentation, and compatibility with patient-derived biofluids. Importantly, such assays should still be interpreted in relation to sample preparation, cohort size, and marker specificity, because plasmonic colorimetric responses can be affected by protein corona formation, ionic strength, and nonspecific adsorption in complex matrices. This recent work strengthens the relevance of plasmonic nanoparticle strategies as simple and potentially clinically deployable exosome-sensing platforms [22].

EVs called tumor-derived exosomes (TEXs) are continually discharged into the bloodstream by tumor cells. TEXs have been suggested as appealing cancer diagnostic indicators by a large body of research. However, because of their extremely low concentrations, TEXs are difficult to detect in blood at early tumor stages. Liu et al. developed a technique known as the PLA-RPA-TMA assay, which enables the very sensitive and specific detection of TEXs. Based on two proximity ligation assay (PLA) probes that identify a biomarker on a TEX, the author produced a distinct surrogate DNA signal for the particular biomarker. This signal was then amplified synchronously twice using transcription-mediated amplification (TMA) in conjunction with recombinase polymerase amplification (RPA), and the RPA-TMA reaction products were quantitatively identified using a colorimetric assay based on gold nanoparticles. Using TEXs from nasopharyngeal carcinoma (NPC) cells, the authors demonstrated proof-of-concept evidence for this method with a detection limit of 10^2^ particles/mL. They also reported measuring plasma Epstein–Barr virus latent membrane protein 1 (LPM1)-positive (LMP1+, accuracy: 0.956) and epidermal growth factor receptor (EGFR)-positive (EGFR+, accuracy: 0.906). TEXs are powerful early NPC diagnostic biomarkers [23].

EVs are a key component of intercellular communication and contain rich molecular information from the parent cell. As a result, they are increasingly being studied in translational medicine. EVs, which include exosomes and microvesicles/microparticles, range in size from 40 nm to 1 μm. They share several physicochemical characteristics with other nano-objects found in bodily fluids, such as single and aggregated proteins, including size, density, surface charge, and light interactions. This makes it difficult and time-consuming to separate, titrate, and characterize EVs. Here, Maiolo et al. introduced a quick and inexpensive colorimetric test that uses the combined effects of colloidal gold nanoplasmonics, nanoparticle–protein corona, and nanoparticle–membrane contact to detect protein contaminants by eye and quantify them in EV preparations. The test has a dynamic range of EV concentrations from 35 fM to 35 pM, which corresponds to the usual range of EV concentrations in bodily fluids, and it reaches a limit of detection (LOD) for protein contamination of 5 ng/μL. The first instance of using the nanoparticle–protein corona in analytical chemistry is shown in this publication [24].

The majority of exosome-analysis techniques used today are laborious and rely heavily on the preisolation step of commercial extraction kits, which necessitates extensive sample manipulation, expensive isolation kits and reagents, and time-consuming procedures, and is prone to bias and artifacts. Here, the authors presented a straightforward technique for the direct separation and subsequent identification of a particular exosome population utilizing gold-loaded ferric oxide nanocubes (Au-NPFe_2_O_3_NC), a designed superparamagnetic material with multifunctional features. To capture the bulk population of exosomes, Au-NPFe_2_O_3_NC were first functionalized with a general tetraspanin (exosome-associated) antibody (i.e., CD63) and then dispersed in the sample fluids, acting as “dispersible nanocarriers.” Au-NPFe_2_O_3_NC-bound exosomes were moved to the tissue-specific, antibody-modified, screen-printed electrode following magnetic collection and purification. Authors employed placenta alkaline phosphatase (PLAP), a particular placental marker, to identify exosomes released by placental cells as a proof of concept. Then, using Au-NPFe_2_O_3_NC’s peroxidase-like activity, an ELISA-based sensing procedure was established for both UV-visible and electrochemical detection of PLAP-specific exosomes in placental cell-conditioned medium. With and without a commercial “total exosome isolation kit”-based preisolation step, the authors showed excellent agreement in analytical performance for the detection of exosomes derived from placental cells (i.e., linear dynamic range, 10^3^–10^7^ exosomes/mL; LOD of 10^3^ exosomes/mL; relative standard deviation (%RSD) of <5.5% for *n* = 3) [25].

Oliveira-Rodríguez et al. effectively developed a unique lateral flow immunoassay (LFIA) for detecting exosomes using tetraspanins as targets. They used this technique to identify exosomes from various sources, including cell culture supernatants, human plasma, and urine. The LFIA technology was tested to reliably count exosomes from a patient’s metastatic melanoma cell line as proof of concept. The test can be completed in 15 min with an LOD of 8.54 × 10^5^ exosomes/mL using a combination of anti-CD9 and anti-CD81 as capture antibodies and anti-CD63 labeled with gold nanoparticles as the detection antibody. Based on the above findings, this platform could be well-suited for rapid exosome quantification, with promising diagnostic applications. However, detecting exosomes from various sources may require adjusting the analytical settings to their specific composition [26].

Wang et al. demonstrated the development of an electrochemical aptasensor for exosome detection using a sandwich-structured nanoporous membrane made by assembling multilayer gold nanoparticles (mulAu) and monolayer gold nanoparticles (monAu) on the opposite side of a nanoporous alumina membrane (NAM). Tumor-derived exosomes can be effectively collected by particular probes located on the monAu side of the membrane. The mulAu side, which was securely bonded to a Si wafer, enabled electrochemical exosome detection by measuring the amperometric signal of ferricyanide as it traveled across the membrane’s nanochannel array. After optimizing the NAM pore width, base sequence of the capture probe, incubation temperature, and exosome incubation period, this NAM-based electrochemical aptasensor was used to quantify MCF-7 cell-derived exosomes. The steady state current showed a linear relationship with exosome concentrations ranging from 10^3^ to 10^7^ particles μL^−1^, with a detection limit of 2.8 × 10^2^ particles μL^−1^. By modifying the capture probe, this sandwich-structured membrane might be used to quantify various tumor-associated vesicles in a label-free, sensitive, and quick manner [27].

(iii) Silver Nanoparticles: The greater plasmonic field enhancement and metal-enhanced fluorescence properties of silver nanoparticles over gold make them scientifically significant for mass magnification. These characteristics minimize steric hindrance and nonspecific adsorption while enabling high signal amplification at lower particle loadings, which is essential for ultrasensitive exosome detection.

**Figure 5:**
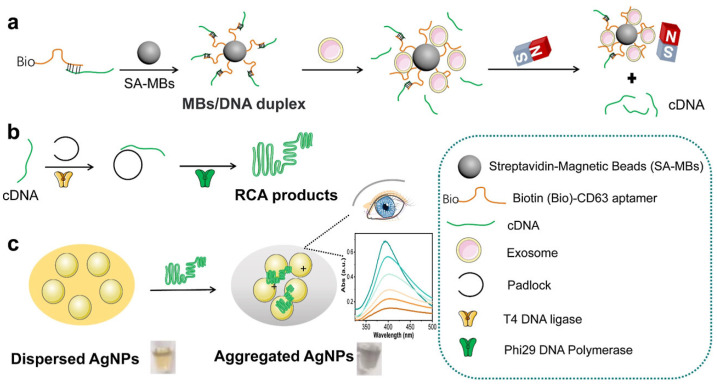
Schematic Illustration of the Process of Exosome Detection; (**a**) Exosome Concentration Converted to the cDNA Evaluation; (**b**) Amplification of the Released cDNA Using RCA; (**c**) RCA Products Detected through AgNP Aggregation by a Colorimetric Method, Visualized by the Naked Eyes or the Quantified Using UV–Vis Spectrometry [28].

Their limitations hamper the clinical application of current detection techniques in terms of sensitivity, specificity, and cost-effectiveness. To overcome these obstacles, Dong et al. have developed a rapid, precise, and cost-effective technique for identifying exosomes with high sensitivity and specificity, making it potentially useful for further clinical evaluation. As a capture probe, streptavidin-coated magnetic beads (SA-MBs) are conjugated to a cluster of differentiation 63 (CD63) aptamer and its complementary DNA (CD63 aptamer/cDNA). By binding to the aptamer and releasing the cDNA, exosomes containing CD63 proteins can start rolling circle amplification (RCA), which amplifies the cDNA copies. Positively charged spermine-modified silver nanoparticles (AgNPs) aggregate as a result of the negatively charged RCA products’ electrostatic attraction. With limits of detection of 4.0 × 10^4^ particles/mL for visual observation and 800 particles/mL for UV-vis spectroscopy, respectively, the aggregation of AgNPs can be visually observed with the unaided eye or quantitatively analyzed using UV-vis spectroscopy to determine the concentration of exosomes. The technique’s potential for clinical application in liquid biopsy has also been established by its ability to identify exosomes in serum samples [28]. Figure 5 illustrates a schematic illustration of the process of exosome detection; (a) exosome concentration converted to the cDNA evaluation; (b) amplification of the released cDNA using RCA; and (c) RCA products detected through the AgNPs aggregation by a colorimetric method, visualized by the naked eyes or quantified using UV–Vis spectrometry.

To find the best metal-enhanced fluorescence (MEF)-based detection with high sensitivity, Goodrum et al. investigated several nanoparticle sizes, shapes, and metal kinds with fluorophores of various wavelengths. Fluorescein isothiocyanate’s (FITC’s) fluorescence intensity was significantly lower than Cy5’s, and the effects of autofluorescence were found to be twice as pronounced. The authors showed how to use the Cy5 fluorophore for multiplexed detection of surface and intravesicular proteins directly from lysed EVs in both buffer and human plasma after choosing 30 nm silver nanoparticles at a concentration of 10^9^ particles mL^−1^. Detection limits in phosphate-buffered saline (PBS) were found to be two to three orders of magnitude lower than those in conventional ELISA. For TGF-β1, AKT1, and TSG101, detection limits of 1.97 × 10^5^ EVs mL^−1^, 1.94 × 10^6^ EVs mL^−1^, and 2.17 × 10^4^ EVs mL^−1^ were obtained directly from human plasma. These findings show that silver nanoparticle-embedded membrane (sNEM) is suitable for highly sensitive, multiplexed detection of EV markers from complicated biofluids for early diagnoses while providing benefits such as ease of use, low reagent/sample consumption, scalability, and reduced sample preparation [29]. Silver nanoparticles primarily contribute through plasmonic and metal-enhanced fluorescence signal amplification; their role in true physical mass amplification is secondary and platform-dependent.

(iv) Hybrid/Heavy Mass-Loading/Signal-Amplifying System Nanoparticles: Applications requiring severe signal amplification through inertial or catalytic mass loading require hybrid mass-loading/signal-amplifying systems, such as metal–semiconductor hybrids and dense nanocomposites. They can detect ultralow-abundance biomarkers in early-stage disease because of their enormous effective mass and multifunctional surfaces, which allow multi-stage amplification (acoustic, catalytic, or electrochemical).
(v) Magnetic Nanoparticles: By enabling active manipulation, enrichment, and preconcentration of exosomes under external magnetic fields, magnetic nanoparticles serve a crucial logistical function. This capacity efficiently increases the local mass density of trapped exosomes and improves downstream sensing sensitivity across different transduction modes by overcoming diffusion-limited transport and matrix interference.

Miao et al. developed a highly sensitive and selective electrochemical biosensor for tumor exosome detection based on a multipedal DNA walker, with the exosome serving as the center of the nanomachine. The method uses magnetic Fe_3_O_4_@Au nanoparticles for separation and a CD63-specific aptamer for targeted recognition. It immobilizes numerous walker DNA strands onto individual exosomes, initiating an autonomous walking process via a three-way DNA junction. This approach significantly amplifies the signal, allowing for an LOD of 6 exosomes/μL and surpassing most existing methods in terms of sensitivity and reproducibility. The methodology exhibits strong selectivity for tumor-derived exosomes over non-tumorigenic exosomes and retains high accuracy even in complex blood samples, highlighting its potential for liquid biopsy applications and expanding the use of DNA nanomachines in cancer diagnosis [30].

Reiner et al. used a grating-coupled surface plasmon resonance (GC-SPR) biosensor to detect tiny lipid EVs with sensitivity. To analyze tiny levels of EVs in complex liquid samples, magnetic nanoparticles are used to pre-concentrate the target analyte on the sensor surface. The affinity binding is then explored using SPR wavelength interrogation. The GC-SPR technique effectively pulls EVs to the sensor surface by using magnetic nanoparticles and an external magnetic field gradient provided by the device. This method overcomes slow diffusion-limited mass transfer and significantly improves sensor response. An SPR sensor chip modified with antibodies against the surface marker CD81 and magnetic nanoparticles binding the vesicles via annexin V and cholera toxin B chain detects various EV populations secreted by mesenchymal stem cells [31].

Huang et al. created unique magnetic graphene oxide nanoparticles (MGONs) for effective exosome capture, in which Fe_3_O_4_@SiO_2_ magnetic nanoparticles were coated with GO via dopamine. MGONs have CD63 aptamers attached to their surfaces, allowing them to identify and bind to CD63 on the exosome membrane. The number of exosomes adsorbed on the MGON surface was 1.5 times that of the Fe_3_O_4_@SiO_2_ surface. Exosome capture rates reached 89.4% at 15 min at room temperature. Furthermore, exosomes may be efficiently enhanced and extracted using an external magnetic field, and the capture technique does not alter their morphological form or biological activity. Using a FAM-labeled single-stranded DNA probe, the fluorescence intensity of collected exosomes was measured, with an LOD of 2.4 × 10^7^ particles/mL. These aptamer-decorated MGONs also displayed high-efficiency exosome capture using cancer patient samples, indicating that the resulting MGONs have great practical relevance in early disease diagnostics and associated clinical applications [32].

(vi) Metal–Organic Framework Nanoparticles: In view of their ultrahigh porosity, huge surface area, and variable coordination chemistry, which enable simultaneous exosome capture and signal molecule loading, MOFs are particularly well-suited for mass magnification. They are ideal for label-free, self-calibrated sensing platforms because of their framework architecture, which enables regulated amplification via guest-molecule confinement while maintaining molecular specificity.

**Figure 6:**
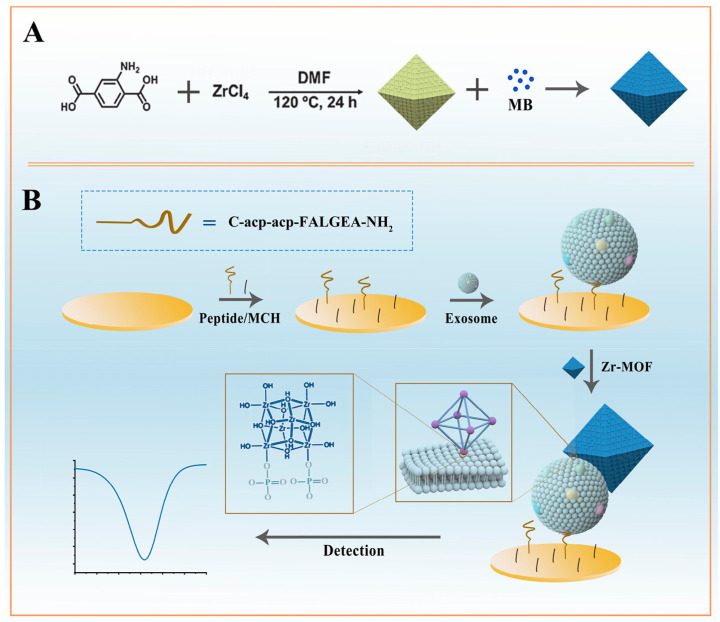
Schematic Diagram of (**A**) the Fabrication Process of MB@UiO-66-Based Nanoprobe and (**B**) the Principle of the Electrochemical Biosensor for the Detection of GBM-Derived Exosomes [33].

Glioblastoma (GBM) is a very deadly brain tumor with complicated oncogenic changes and a blood–brain barrier that makes early identification difficult. GBM-derived exosomes can pass the blood–brain barrier and circulate in bodily fluids, making them noninvasive indicators that contribute to early-detection assay development. Sun et al. developed a label-free electrochemical biosensor using Zr-based metal–organic frameworks (Zr-MOFs) to detect GBM-derived exosomes, with practical applications. A peptide ligand is designed to connect with EGFR and EGFRvIII, which are overexpressed on GBM-derived exosomes. Zr-MOFs encapsulated with methylene blue can absorb on exosome surfaces by interacting with Zr^4+^ and intrinsic phosphate groups outside the exosome. The concentration of exosomes can be directly detected by monitoring electroactive molecules inside MOFs, with a detection limit of 7.83 × 10^3^ particles/μL. This biosensor has the potential to distinguish between GBM patients and healthy groups, indicating it may support future diagnostic development [33]. Figure 6 presents a schematic diagram of (A) the fabrication process of the MB@UiO-66-based nanoprobe and (B) the principle of the electrochemical biosensor for the detection of GBM-derived exosomes.

Exosomes are noninvasive indicators that play a significant role in cancer screening, immunological response, and physiological processes. Detecting cancer cell-derived exosomes accurately is crucial for early cancer diagnosis in patients. This study created a self-calibrating aptasensor for exosomes using a hybrid thin-film platform. The platform was built by assembling black phosphorus nanosheets (BPNSs) and ferrocene (Fc)-doped metal–organic frameworks (ZIF-67) on an indium tin oxide (ITO) slice, then adding a methylene blue (MB)-labeled single-strand DNA aptamer to the ITO slice. The aptamer-BPNSs/Fc/ZIF-67/ITO platform demonstrated dual redox-signal responses for MB (labeled on the aptamer) and Fc (doped into ZIF-67). A self-calibrated aptasensor was developed for sensitive detection of exosomes, with an LOD as low as 100 particles mL^−1^. The aptasensor accurately detects cancer cell-derived exosomes in serum and plasma samples from both healthy and breast cancer patients. This aptasensor has great performance and can detect many biomarkers from cell line exosomes. It is useful for developing new approaches for detecting exosomes from various cancer cells. This work aims to improve strategies for early cancer screening and detection [34].

Ni et al. described a dual MOF-based electrochemical biosensor that can directly capture and identify malignant exosomes in complicated biological samples. This biosensor consists of three main components: a pH-sensitive Zeolitic imidazolate framework-8 (ZIF-8)-engineered screen-printed carbon electrode for signal transduction, a pH-insensitive magnetic nanoparticle-modified copper-based MOF (MNP/Cu-BTC MOF) for exosome collection, and a DNA logic device for DNA computation. MNP/Cu-BTC MOF can directly and quickly extract exosomes from biological media, eliminating the need for costly antibodies or time-consuming centrifugation methods. Using a DNA-based logic system, streptavidin–glucose oxidase (SA-GOx) may be chemically attached to the surface of exosomes via the biotin-streptavidin contact. This biosensor enables ultrasensitive detection of exosomes by combining magnetic enrichment, acidification of the fluid around the electrode via enzymatic conversion of glucose to gluconic acid, and ZIF-8-mediated signal transduction. The biosensor can detect exosomes at a label-free LOD of 2.2 × 10^4^ particles mL^−1^. The suggested biosensor, with its ability to easily and accurately collect, identify, and sensitively detect exosomes, has the potential to accelerate the translation of exosome analysis into routine translational use after further validation [35].

(vii) Metal Oxide Nanoparticles: Due to their high surface reactivity, enzyme-like catalytic activity, and chemical stability, metal oxide nanoparticles provide strong mass magnification. Their capacity to participate in surface charge exchanges and redox processes enables reliable signal amplification even in challenging biological contexts, supporting scalable, repeatable clinical diagnostics.

**Figure 7:**
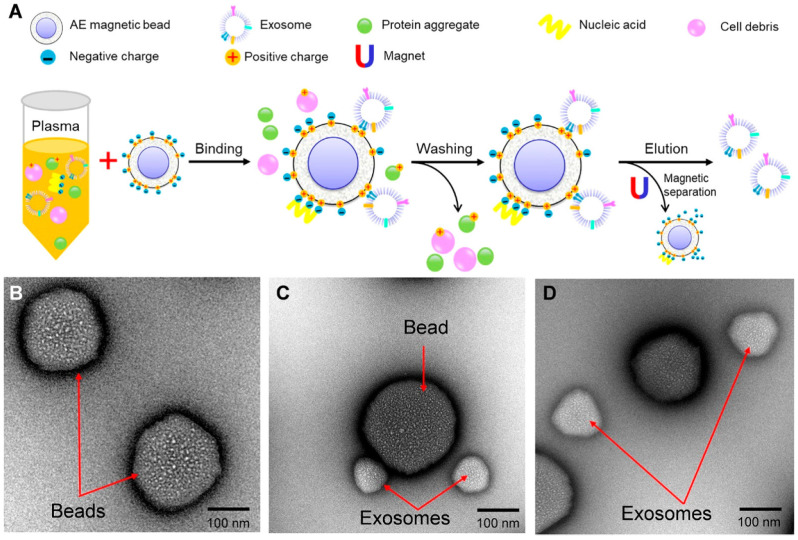
Schematic of the AE-based isolation of exosomes. (**A**) Diagram of AE-based isolation of exosomes. (**B**) TEM characterization of AE magnetic beads. (**C**) TEM characterization of AE magnetic beads after capture of exosomes. (**D**) TEM characterization of AE magnetic beads and exosomes after elution [36].

In this paper, Chen et al. presented an anion exchange (AE)-based isolation method that was first proposed to isolate exosomes directly from plasma and cell-culture medium with AE magnetic beads within 30 min. Exosomes isolated with AE magnetic beads had higher recovery efficiency (>90%) and fewer protein impurities than those isolated by ultracentrifugation (UC). Prostate cancer (PCa) exosomes in plasma were detected in a visual, label-free, and quantitative manner with aptamer-capped Fe_3_O_4_ nanoparticles for the first time. The linear range of PCa exosomes was estimated from 0.4 × 10^8^ to 6.0 × 10^8^ particles/mL with a detection limit of 3.58 × 10^6^ particles/mL. The present study provides an efficient and practical approach for the rapid isolation and visible detection of exosomes, which is promising for contributing to early-detection assay development of PCa [36]. Figure 7 describes the schematic of the AE-based isolation of exosomes.

Herein, Chen et al. presented a zinc oxide (ZnO) nanowire-coated three-dimensional (3D) scaffold chip device for effective immunocapture and classically visible and colorimetric detection of exosomes. The chip device is composed of a 3D polydimethylsiloxane (PDMS) scaffold skeleton covered by a free-standing ZnO nanowire array. The interconnected micropores of the 3D scaffold induce the fluid flow with chaotic or vortex features, and the ZnO nanowire array provides a large surface area for immobilization of exosome-specific antibody, as well as a size exclusion-like effect for retaining exosomes. These synergistically and significantly enhance the capture of exosomes at a high flow rate. The captured exosomes are detected by a horseradish peroxidase (HRP)-labeled antibody, which can initiate 3,3′,5,5′-tetramethylbenzidine (TMB)-based colorimetric sensing. The quantitative readout of exosomes is easily accomplished by UV–vis spectrometry or a microplate reader with a linear range of 2.2 × 10^5^ to 2.4 × 10^7^ particles/μL and a minimal detectable concentration of 2.2 × 10^4^ particles/μL. This chip device was applicable to clinical samples where cancer patients demonstrate a statistically significant increase in exosomes compared with healthy individuals. Thus, the chip device is cost-effective and easy to use, facilitating visible and colorimetric assays with high sensitivity toward clinical applications [37].

Chen et al. presented a sandwich-type fluorescent biosensor for the determination of tumor-related exosomes. It is based on magnetic nanoparticle (MNP) capture and HRP catalysis. MNPs were used as the substrate to capture exosomes by modifying the CD63 antibody on the MNPs’ surface. After that, the biotinylated epithelial cell adhesion molecule (EpCAM) antibody was used to capture the tumor-related exosomes, which specifically express EpCAM. A novel method for the fluorescence measurement of tumor-associated exosomes was achieved, with an LOD as low as 200 (±9) particles mL^−1^. The analytical range of this method is from 576 (±15) particles mL^−1^ to 5.76 × 10^7^ (±5.1 × 10^5^) particles mL^−1^. This sensor was also able to successfully detect the exosomes from the plasma of patients with hepatocellular carcinoma (HCC) and healthy humans [38].

This study, done by Shi et al., presents a fluorometric method using magnetic nanoparticles and the hybridization chain reaction to detect exosomes from HepG2 cells with great sensitivity. The surface of magnetic nanoparticles was changed to include antibodies as a recognition element. An antibody was employed to capture the exosome. Probe 1 consisted of an aptamer sequence and a trigger sequence. The aptamer sequence interacts with the surface protein of exosomes. The trigger sequence will hybridize with probes 2 (FAM-labeled) and 3 (FAM-labeled). The product of the hybridization chain reaction will emit a strong fluorescence signal. The hybridization chain reaction produces fluorescence, which is linked to magnetic nanoparticles via the ‘magnetic nanoparticles–antibody–exosome–aptamers’ structure. The presence of magnetic nanoparticles allows the product to be separated from the matrix. The excitation was adjusted to 490 nm. The fluorescence value of the emission spectra at 519 nm was used as the signal response. The linear range of this test is 1000 to 10^7^ particles × mL^−1^. The LOD was 100 particles × mL^−1^, and it was used to determine exosomes from hepatic cancer cells [39].

He et al. developed a low-cost assay to capture and identify exosomes using a copper-mediated signal amplification method. The assay has three steps. To catch bulk nanovesicles, cholesterol-modified magnetic beads (MBs) interact hydrophobically with lipid membranes. Exosomes with specified membrane proteins were attached using aptamer-modified CuO NPs to produce sandwich complexes (MB–exosome–CuO NP). Acidolysis dissolves CuO NP into Cu(II) ions (Cu^2+^), which can then be reduced to fluorescent copper nanoparticles (CuNPs) with sodium ascorbate and poly(thymine). CuNPs fluorescence emission increased with Cu^2+^ concentration and is proportional to exosome concentration. The authors’ approach analyzed exosomes in biological samples at concentrations ranging from 7.5 × 10^4^ to 1.5 × 10^7^ particles/μL, with an LOD of 4.8 × 10^4^. The overall working time was approximately 2 h. The assay was a simple and cost-effective way to analyze exosomes in biological samples [40].

Kuang et al. demonstrated that the nanozyme developed in this study incorporates polydopamine-functionalized graphene oxide (rGO@PDA) units that adsorb ssDNA aptamers, as well as CeO_2_ metal oxide nanosheet units with peroxidase activity. Adenosine triphosphate (ATP) acts as an auxiliary regulator, increasing the peroxidase activity of rGO@PDA@CeO_2_ throughout a wide pH range (4–7.4), breaking the constraint that typical nanozymes can only display peroxidase activity under acidic circumstances. The ssDNA aptamers, which are exosome recognition probes, may be adsorbed on the surface of rGO@PDA by aromatic stacking, competing with ATP molecules. Thus, the ssDNA aptamer serves as a switch for a colorimetric sensor, allowing it to modulate the peroxidase activity of the rGO@PDA@CeO_2_ under physiological settings and identify exosomes with high sensitivity. The test has an LOD linear range of 0.46 × 10^7^–10^8^ particles/mL and a limit of 3.81 × 10^5^ particles/mL. Furthermore, this aptasensor detects six exosomal proteins across five cell types and clinical samples, indicating its potential for clinical diagnostics [41].

(viii) Titanium Nitride Nanomaterials: Titanium nitride nanoparticles offer improved chemical and thermal stability together with plasmonic activity, making them a scientifically required substitute for noble metals. For real-time and long-term exosome monitoring, its interoperability with label-free surface plasmon resonance sensors allows physical mass amplification without sacrificial labels.

**Figure 8:**
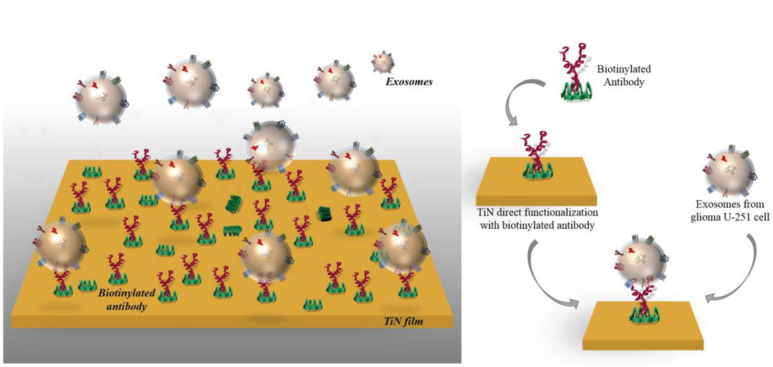
Schematic illustration of TiN functionalized by biotinylated anti-CD63 antibody for the detection of U251 GM-derived exosomes [42].

Qiu et al. proposed that titanium nitride (TiN) is an excellent alternative to gold and silver for plasmonic support, with tunable capabilities in the visible and near-infrared spectrum. However, label-free surface plasmon resonance biosensing using TiN is rarely reported due to inadequate surface functionalization procedures. This study presents biotinylated antibody-functionalized TiN (BAF-TiN) for high-performance label-free biosensing applications. The BAF-TiN biosensor detects 30–200 nm exosomes from a human glioma cell line. The BAF-TiN biosensor has an LOD of 4.29 × 10^−3^ μg mL^−1^ for exosome marker CD63 and 2.75 × 10^−3^ μg mL^−1^ for epidermal growth factor receptor variant-III, a glioma-specific mutant protein. The BAF-TiN biosensor, with its biocompatibility, high stability, and label-free sensing capabilities, has the potential to detect cancer biomarkers such as exosomal surface proteins [42]. Figure 8 shows the schematic illustration of TiN functionalized by biotinylated anti-CD63 antibody for the detection of U251 GM-derived exosomes.

Disposable plasmonic metasurfaces with excellent biosensing capability are urgently required for clinical label-free detection. Low-cost aluminum (Al) and TiN are viable alternatives to noble metals for creating these metasurfaces. However, Al has low chemical stability, and TiN has modest plasmonic effects, which make them unsuitable for meta-biosensing applications. Li et al. use their complementary advantages to offer TiN/Al meta-biosensors. They not only enable the unique near-field sensing enhancement via TiN/Al hybrid plasmonic modes but also provide a strong TiN armor to protect against external wear, heat, moisture, and corrosion throughout the bio-detection process. Compared to typical gold-based sensors, the meta-biosensors provide higher optical sensitivity at lower cost and with fewer pretreatment steps. The strong biosensing performance enables the construction of a high-throughput detection method for serum small extracellular vesicles (sEVs), which aids in the diagnosis and monitoring of prostate cancer. The sEV meta-biosensing has a diagnostic sensitivity of 100% for differentiating early cancer, breaking beyond traditional testing limitations. Furthermore, it improves the accuracy of predicting cancer recurrence risk after surgery. Their findings demonstrated the feasibility of large-scale production of strong meta-biosensors based on non-noble materials, opening new avenues for cancer detection and prognosis [43].

(ix) Upconversion Nanomaterials: Although their near-infrared excitation lowers autofluorescence and scattering, upconversion nanomaterials are essential for mass magnification in optically noisy biological systems. They enable high-contrast signal amplification that is directly proportional to exosome concentration by transforming low-energy excitation into high-energy emission.

Upconversion nanomaterials are inorganic nanoparticles doped with rare-earth ions. Because of their near-infrared excitation characteristic, they may efficiently prevent interference from autofluorescence and scattered light from the biological sample itself, as well as photodamage induced by UV or visible light stimulation. Upconversion nanomaterials are also effective resonant energy donors.

For the easy detection of exosomes, Chen et al. employed a simple paper-supported aptasensor based on luminescence resonance energy transfer (LRET) from upconversion nanoparticles (UCNPs) to gold nanorods (Au NRs). When exosomes are present, the two parts of the aptamer can interact with the CD63 protein on the exosome surface to produce a conjugation that closes the distance between UCNPs and Au NRs, therefore initiating the LRET and promoting luminescence quenching. The self-imaging system can monitor these fluctuations, and the green channel intensities of the resulting colored pictures were retrieved using Photoshop software to measure the luminescence. The luminescence of UCNPs is linearly connected to the quantity of exosomes (1.0 × 10^4^~1.0 × 10^8^ particles/μL), allowing for identification and measurement. Using UCNPs as a luminous material, this technique can detect exosomes at a low detection limit (1.1 × 10^3^ particles/μL) while reducing the background signal. This work presents an effective and practical method for detecting exosomes, which might lead to point-of-care testing in clinical settings [44].

Wang et al. suggested a washing-free aptasensor based on LRET between rare-earth-doped UCNPs donor and tetramethyl rhodamine (TAMRA) acceptor for extremely sensitive exosome detection. The combination of UCNPs and TAMRA can be done by connecting aptamers to the epithelial cell adhesion molecule EpCAM, which is one of the most highly expressed surface proteins of exosomes. As a result, when the UCNPs-TAMRA system in the presence of exosomes is activated by near-infrared light at 980 nm, TAMRA produces yellow fluorescence at 585 nm owing to LRET. As a result, the fluorescence intensity at 585 nm is linearly associated with exosome content, making it possible to identify and quantify exosomes. Under optimum conditions, the suggested technique may achieve a low LOD of 80 particles/μL and efficiently decrease background signal by employing UCNPs as an energy donor. Furthermore, by carefully screening the aptamers, this suggested aptasensor may be used to detect various targets worldwide [45].

**Figure 9:**
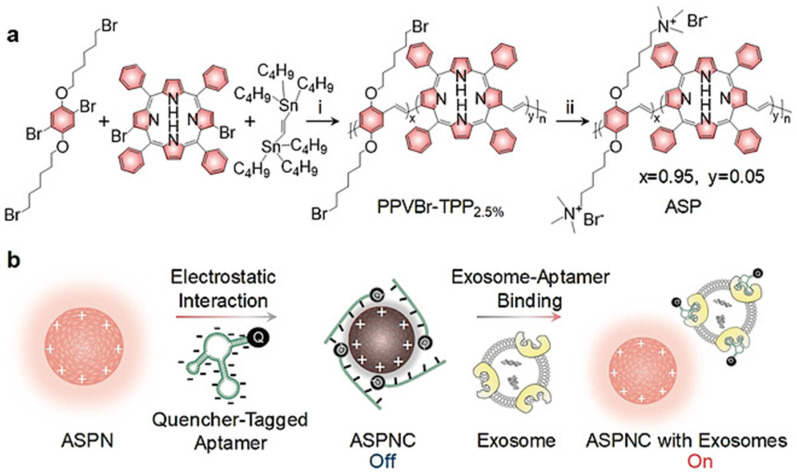
Design and sensing mechanism of ASPNC. (**a**) Synthetic route of ASP. Reagents and conditions: (i) tris(dibenzylideneacetone)dipalladium(0) [Pd_2_(dba)_3_], tri(p-tolyl)phosphine (TPTP), chlorobenzene, 100 °C, 24 h; (ii) trimethylamine, tetrahydrofuran (THF), methanol, 24 h. (**b**) Illustration of the formation of ASPNC and the afterglow detection of exosomes [46].

Lyu et al. created the first luminous nanosensor for multiplex differentiation of cancer exosomes that does not require real-time light excitation. The sensor is made of a near-infrared semiconducting polyelectrolyte (ASPN) that interacts with a quencher-tagged aptamer. The afterglow signal of the nanocomplex (ASPNC), which was originally suppressed, is activated in the presence of aptamer-targeted exosomes. Because afterglow detection occurs after excitation, background signals are reduced, resulting in a detection limit that is roughly two orders of magnitude lower than fluorescence detection in cell culture conditions. Also, ASPNC may be readily modified to detect different exosomal proteins by modifying the aptamer sequence. This permits an orthogonal study of several exosome samples, possibly providing an accurate identification of the cellular origin of exosomes for cancer detection [46]. Figure 9 shows the design and sensing mechanism of ASPNC.

UCNP-based LRET biosensing has several benefits, including wash-free detection and accurate biomolecule quantification. However, its sensitivity is limited by the continuous energy transfer in co-doped UCNPs during LRET. Kim et al. reported a time-gated LRET method employing NIR long-lived luminous UCNP donors (L-TG-LRET), which resulted in an 8-fold increase in luminescence lifetime while maintaining emission intensity. This delayed energy migration and transfer mechanism improves sensitivity by inhibiting quick Tm^3+^ reactivation during LRET to IRDye800 acceptors. Using this technique to identify microRNAs (miRNAs), the authors achieved a 17.9-fold greater sensitivity than standard steady-state approaches. Furthermore, the L-TG-LRET can accurately quantify miRNA expression in cancer cells, plasma, and exosomes, allowing cancer patients to be distinguished from healthy donors. Notably, this method beats polymerase chain reaction in identifying low-abundance exosomal miRNAs. These findings demonstrate the L-TG-LRET system’s potential as a useful tool for sensitive biomolecular detection in clinical diagnostics [47].

(x) Zirconium–Phosphate Nanoparticles: By taking advantage of intrinsic phosphate groups on exosome membranes, zirconium–phosphate coordination chemistry presents a chemically orthogonal amplification technique. This method reduces assay complexity while preserving excellent sensitivity by enabling enzyme-free, label-free mass magnification through vesicle–vesicle bridging.

Zr-MOFs were used by Wang et al. to identify EpCAM-positive exosomes in breast cancer. Because Zr-MOFs, like uiO-66, have a strong affinity for phosphate groups, they may selectively enrich phosphate molecules and nucleic acids by forming Zr-O-P interactions. Furthermore, the fluorescence characteristics of uiO-66-NH_2_ particles are good. Based on these ideas, uiO-66 (uiO66@Fe_3_O_4_) was magnetically modified to function as an adsorbent, allowing for the quick enrichment and capture of exosomes by phosphate anchoring. To identify isolated EpCAM-positive exosomes, a fluorescent probe was created utilizing uiO-66-NH_2_ decorated with anti-EpCAM antibodies (µiO-66-NH_2_@anti-EpCAM), which produced a “MOF–exosome–MOF” structure. The technique showed an LOD of 16.72 particles/µL [48].

Wang et al. described a novel and elegant strategy for exosome detection that employs the unique zirconium–phosphate coordination chemistry to form a sandwich structure that connects exosomes and liposomes. The authors create a label-free, cost-effective, and sensitive analytical platform by taking advantage of the inherent phosphate groups found in the phospholipid bilayers of exosomes and liposomes. The method captures exosomes from complex materials using CD63 antibody-functionalized magnetic beads. Zr^4+^ ions are then used to cross-link them with calcein-encapsulated liposomes. When liposomes are lysed, the emitted calcein fluorescence acts as a direct readout for exosome measurement. The authors show that the Zr^4+^-mediated system generates signals with excellent specificity, as no fluorescence is detected without Zr^4+^ or exosomes. The technique has a high LOD of 7.6 × 10^3^ particles/mL, a wide linear dynamic range, and reliable performance in the presence of a biological matrix (10% FBS). Furthermore, the procedure is straightforward, does not involve any chemical modification of biological targets, and the utilization of liposomes allows for effective signal amplification without the requirement for enzymes or multistep labeling. This test differs from existing approaches in that it is simple, sensitive, and potentially adaptable to a wide range of phospholipid-containing vesicles or analytes. Overall, the study not only introduces a revolutionary biosensor platform for exosome detection, but it also opens up new avenues for biosensor creation using coordination chemistry, making it an important contribution to analytical and biomedical sciences [49].

**II. Nonmetallic Nanoparticles**: Alternative to using pure mass density, nonmetallic nanomaterials offer an alternative amplification paradigm based on electronic conductivity modulation, catalytic mimicry, and nanoscale charge transfer. Quantum-confined nanostructures, two-dimensional semiconductors, and carbon-based materials provide tunable electronic states, rich surface chemistry, and extraordinarily large surface areas that effectively pair with biological recognition events.

Nonmetallic nanoparticles often serve as intrinsically amplified signal transducers in mass magnification techniques, where binding-induced alterations propagate through conductive or catalytic networks. In contrast to dense metals, these materials provide highly sensitive detection even at low absolute mass loading by amplifying signals through variations in electron transfer rates, luminescence efficiency, or catalytic turnover.

(i) Carbon Nanomaterials: Through their intrinsic catalytic mimics, variable surface chemistry, and high electrical conductivity, carbon-based nanomaterials are scientifically crucial for mass magnification. These characteristics enable exosome-binding events to spread via electron-transfer networks, boosting signals without the need for large metallic labels.

Due to its large surface area, exceptional flexibility, and high porosity, mesoporous carbon, a novel class of three-dimensional, conductive carbon materials, has garnered increased interest. Sahraeia et al. demonstrated in this study how a very flexible chromatography paper with a lab-made carbon ink was used to create an affordable, biodegradable electrochemical sensor. Mesoporous carbon functionalized with gold nanoparticles (AuNPs@MCF/MWCNTs) and multi-carbon nanotubes improved the electrochemical response. After that, the CD9 antibodies were immobilized on the nanocomposite’s porous surface so they could bind to proteins that were overexpressed on the exosomal membrane. This structure and electrostatic repulsion efficiently shield the redox probe’s electron transport, allowing for the quantitative identification of exosomes through current variations. With a linear range of 1 × 10^2^ to 1 × 10^7^ exosomes/µL and a very low LOD of 70 exosomes/µL, this paper-based immunosensor demonstrated good sensitivity and appropriate selectivity for the detection of exosomes in the plasma sample. This paper-based immunosensor can pave the way for the use of this type of biosensor in accessible, compact systems, particularly point-of-care testing (POCT) devices, for the early detection and screening of illnesses such as cancer [50].

**Figure 10:**
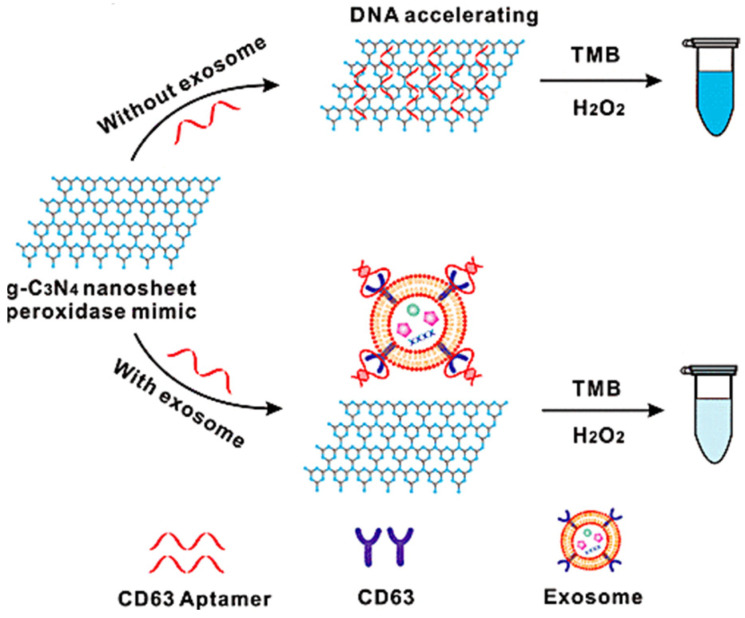
Illustration of DNA Aptamer Accelerating the Intrinsic Peroxidase-Like Activity of g-C_3_N_4_ NSs for the Detection of Exosomes [51].

Wang et al. found that single-stranded DNA (ssDNA) can enhance the intrinsic peroxidase-like activity of g-C_3_N_4_ nanosheets (NSs). The authors discovered that ssDNA adsorbed on g-C_3_N_4_ nanosheets enhances their catalytic activity. The ssDNA-NSs hybrid accelerated H_2_O_2_-mediated TMB oxidation by at least four times compared to unmodified NSs. The increased activity can be attributed to the strong connection between TMB and ssDNA, which is mediated by electrostatic attraction and aromatic stacking, as well as the length and base composition of the latter. The ssDNA-NSs hybrid’s high catalytic activity enabled sensitive colorimetric detection of exosomes when an aptamer against CD63, a surface marker of exosomes, was used in hybrid formation. The sensor detected CD63 expression differences in exosomes from the breast cancer cell line (MCF-7) and the breast cell line (MCF-10A). Circulating exosomes from breast cancer patients and healthy controls showed a similar trend. The study suggests that ssDNA can boost the peroxidase activity of nanomaterials and has considerable potential for clinical diagnosis using liquid biopsy [51]. Figure 10 presents an illustration of DNA aptamer accelerating the intrinsic peroxidase-like activity of g-C_3_N_4_ NSs for the detection of exosomes.

Xia et al. developed a visible and simple method for detecting exosomes using single-walled carbon nanotubes (s-SWCNTs) with exceptional water solubility and an aptamer. Aptamers, specific to exosomes transmembrane protein CD63, are absorbed onto the surface of s-SWCNTs. This improves the mimic peroxidase activity of s-SWCNTs, which can efficiently catalyze the oxidation of TMB, resulting in a colorless to blue solution. Exosomes bind aptamers with CD63, causing conformational changes on the surface of s-SWCNTs. This causes the hue of the solution to shift from deep to moderate, which can be detected visually and measured by UV-vis spectrometry. Exosomes have a linear range of 1.84 × 10^6^ to 2.21 × 10^7^ particles/μL, with an LOD of 5.2 × 10^5^ particles/μL. A visible and simple way of detecting exosomes was successfully developed. The suggested colorimetric aptasensor can detect several targets by simply changing the aptamer [52].

Through orthogonal, label-free detection techniques, recent research has further demonstrated the significance of 2D carbon-based nanomaterials in exosome sensing. For the purpose of differentiating, and fibroblast-derived exosomes, Gil et al. created a graphene-based field-effect transistor (GFET) platform combined with surface-enhanced Raman scattering (SERS). Graphene functioned as both an electrically active transducer and a tag-free Raman-active diagnostic template in this design, allowing for the simultaneous extraction of optical fingerprints from biomolecular Raman bands and electrical features like resistance changes and Dirac point shifts. Crucially, when electrical stimulation was used during Raman acquisition, the combination of FET and SERS outputs greatly enhanced categorization performance as compared to either modality alone, reaching an overall accuracy of 93%. In addition to being extremely sensitive transducers for exosome capture analysis, this work shows that graphene and similar 2D materials are potent multimodal platforms for reliable, label-free cancer liquid biopsy [53].

Zhang et al. developed a sensitive electrogenerated chemiluminescence (ECL) biosensor to detect exosomes. They used aptamer-modified Ti_3_C_2_ MXenes nanosheets as the ECL nanoprobe due to their large surface area, excellent conductivity, and catalytic properties. EpCAM protein-recognized aptamers can efficiently capture exosomes on electrode surfaces. The ECL nanoprobe can identify exosomes and improve luminol ECL signals. This technique yielded a highly sensitive ECL biosensor for detecting MCF-7 cells exosomes. The LOD of 125 particles μL^−1^ was over 100 times lower than that of traditional ELISA methods. The constructed ECL biosensor successfully detected MCF-7 exosomes in serum. This concept offers a practical, sensitive, and dependable method for detecting exosomes in clinical diagnostics [54].

In this effort. Zhang et al. created an ECL biosensor for exosomes and their surface proteins using gold nanoparticles (AuNPs) and Ti_3_C_2_ MXenes hybrids with aptamer modification (AuNPs–MXenes–Apt). This technique successfully caught exosomes using a modified electrode interface with a CD63 aptamer that recognizes exosomes. Gold nanoparticles were formed in situ on single-layer Ti_3_C_2_MXenes with aptamer (MXenes-Apt) modification. MXenes served as both a reductant and stabilizer, eliminating the need for additional reductants or stabilizers. In situ formed AuNPs–MXenes–Apt hybrids effectively recognize exosomes and provide a catalytic surface with high electrocatalytic activity. Gold nanoparticles with predominated (111) facets significantly improved luminol’s ECL signal. A highly sensitive ECL biosensor for detecting exosomes was developed using AuNPs–MXenes–Apt due to its huge surface area, good conductivity, and catalytic properties. The LOD for exosomes produced from the HeLa cell line was 30 particles μL^−1^, which is over 1000 times lower than the usual ELISA approach. The linear range was from 10^2^ to 10^5^ particles μL^−1^. The ECL sensing platform was highly selective for exosomes and their surface proteins from various tumor cell lines (HeLa, OVCAR, and HepG2), allowing for sensitive and accurate detection of exosomes in human serum. This suggests that the ECL biosensor is a reliable tool for exosome-related clinical diagnostics [13].

Zhang et al. used g-C_3_N_4_ conjugated polydopamine-coated Galinstan liquid metal shell-core nanohybrids (g-C_3_N_4_@Galinstan-PDA) nanoprobes and a multivalent PAMAM-AuNPs electrode interface to detect exosomes and their surface proteins using an ECL biosensor. The antibody-modified PAMAM-Au nanoparticles (NPs) electrode interface effectively captures exosomes through multiple recognition mechanisms. Meanwhile, Galinstan nanoparticles were used as the nanoprobe. The antibody-modified g-C_3_N_4_@Galinstan-PDA can detect exosomes and provide sustained ECL signals due to Galinstan NPs’ ability to facilitate electron transfer and prevent g-C_3_N_4_ passivation during electrochemical reduction operations. The study of HeLa cell-derived exosomes achieved remarkable sensitivity with an LOD of 31 particles/μL. The scientists analyzed exosomes in serum, urine, and blood samples and found biomarkers (GPC1, CD9, CEA, and AFP) on exosome surfaces from various cell lines (HeLa, OVCAR-3, and BT474). The suggested ECL biosensor has the potential to be a valuable tool for exosome research, clinical diagnosis, and wearable technologies [55].

Feng et al. developed a novel ECL aptasensor for the ultrasensitive detection of tumor-derived exosomes using a DNA walking machine. Exosomes, tiny vesicles released by cells that transport molecular cargo such as proteins and nucleic acids, are emerging as important participants in intercellular communication and have great potential as biomarkers for cancer detection. The sensor uses a DNA walking machine, which is activated by the binding of a CD63-specific aptamer to exosomes, resulting in autonomous strand displacement and signal amplification on an electrode surface. This technique produces a measurable ECL quenching effect in proportion to exosome concentration, allowing for quantitative study. The technique is very sensitive, with an LOD of about 60 particles/μL, and has a high selectivity for various cell-derived exosomes. The merging of aptamer-based recognition with DNA nanotechnology presents a promising platform for the precise and sensitive detection of exosomes, boosting the potential for early cancer diagnosis and monitoring [56].

(ii) Quantum Dot Nanoparticles: Through combining high surface area with quick charge transport and catalytic activity, two-dimensional nanomaterials like MXenes allow for effective physical mass amplification. Dense exosome capture is made easier by their planar design, which also amplifies luminescent and electrochemical signals with low diffusion barriers.

Boriachek described a stripping voltammetric immunoassay for the electrochemical detection of disease-specific exosomes that utilized quantum dots as signal amplifiers. The test consists of three steps: first, bulk exosome populations are magnetically collected on magnetic beads using a general tetraspanin antibody (e.g., CD9 or CD63), and then disease-specific exosomes are identified using cancer-related. The authors employed CdSe quantum dot (CdSeQD) functionalized biotinylated HER-2 and FAM134B antibodies as breast and colon cancer indicators. Following magnetic washing and purification, acid dissolution of CdSeQDs and anodic stripping voltammetric measurement of Cd^2+^ were performed at the bare glassy carbon working electrode. This approach accurately detected 100 exosomes/μL with a %RSD of <5.5% in cancer cell lines and a small cohort of serum samples (*n* = 9) from colorectal adenocarcinoma patients [57].

**Figure 11:**
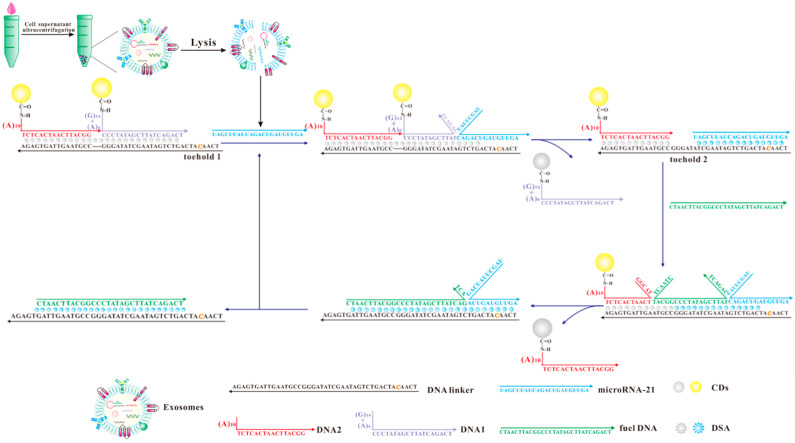
Schematic Representation of the Detection Mechanism of the Proposed Method for Exosomal miRNA-21 [58].

Xia developed a ratiometric fluorescent bioprobe that detects exosomal miRNA-21 using DNA-labeled carbon dots (DNA-CDs) and 5,7-dinitro-2-sulfo-acridone (DSA) coupled with signal amplification for the target. When the bioprobe was built, the fluorescence resonance energy transfer (FRET) between carbon dots (CDs) and DSA was extremely efficient. However, in the presence of the target and after dismantling the fluorescent bioprobe, the fluorescence intensities of CDs and DSA altered synchronously. Because of the ratio of dual fluorescence intensities, this ratiometric fluorescent bioprobe was able to balance out environmental changes by measuring the emission intensity ratio at two separate wavelengths, making it robust and steady enough to identify exosomal miRNA-21. Furthermore, the scientists demonstrated that a single miRNA-21 may potentially accelerate the disintegration of many CDs via DSA, resulting in a considerable shift in the fluorescence ratio for miRNA-21 detection. With this signal amplification approach, the detection limit was as low as 3.0 fM. Furthermore, the inclusion of lock nucleic acid to facilitate the strand displacement process significantly increased the strategy’s selectivity, even against single base mismatch sequences. More crucially, the technique may track the dynamic changes in exosomal miRNA-21, potentially serving as a tool for distinguishing cancer exosomes from nontumorigenic ones. In a nutshell, the ratiometric fluorescent bioprobe demonstrated exceptional stability, sensitivity, and selectivity while being simple to use and cost-effective [58]. Figure 11 shows a schematic representation of the detection mechanism of the proposed method for exosomal miRNA-21.

Boriachek described an electrochemical method for detecting cancer-derived exosomal miRNAs in human blood samples, which involves selectively separating the target miRNA using magnetic beads pre-functionalized with capture probes and then directly adsorbing the targets onto a gold electrode surface. Electrochemical detection of adsorbed miRNA is performed using a 4−/3− redox system. This approach has an outstanding detection sensitivity of 1.0 pM and a relative standard deviation (%RSD) of <5.5% in cancer cells and serum samples (*n* = 8) taken from patients with colorectal adenocarcinoma [59].

Dong et al. created a quick way to quantify EVs using a lateral flow test and membrane biotinylation approach. Using biotin-functionalized phosphatidylethanolamine (DSPE-PEG-Biotin), the membrane of EVs was successfully changed with biotin under strong hydrophobic interactions. To quantify the strong affinity between streptavidin and biotin, a lateral flow experiment was used with fluorescent nanospheres (FNs) as a reporter. Biotinylation of biogenic EVs could be as high as 85%. The suggested approach accurately detects 2.0 × 10^3^ particles/μL. The entire treatment took approximately one hour. Additionally, this method was used to detect EVs in biological samples, highlighting potential clinical applications [60].

Qiao et al. described an ECL aptasensor that detects exosomes from breast carcinoma cells. Mercaptopropionic acid (MPA)-modified Eu^3+^-doped CdS nanocrystals (MPA-CdS:Eu nanocrystals (NCs) and H_2_O_2_ served as ECL emitters and coreactants, respectively. The CD63 aptamer recognizes and captures exosomes, forming a G-quadruplex/hemin DNAzyme that rapidly decomposes H_2_O_2_. This results in a diminished ECL signal of MPA-CdS:Eu NCs. MCF-7 cells produce exosomes at concentrations ranging from 3.4 × 10^5^ to 1.7 × 10^8^ particles/mL. An LOD was determined to be 7.41 × 10^4^ particles/mL with a signal-to-noise ratio of 3. The aptasensor has effectively detected exosomes in serum [61].

Fang et al. developed an ECL and photothermal dual-mode biosensor to detect exosomes using black phosphorus (BP) quantum dots (BPQDs) and MXenes as signal amplifiers. BPQDs can catalyze the oxidation of Tris (4,4′-dicarboxylicacid-2,2′-bipyridyl) ruthenium(II) dichloride Ru(dcbpy)_3_^2+^ and were employed as a coreactant for the first time. The self-enhanced Ru(dcbpy)_3_^2+^@BPQDs ECL system produces a strong ECL signal by minimizing energy loss and electron transfer distance. MXenes, with their high specific surface area and conductivity, were used as a support to augment the immobilization of Ru(dcbpy)_3_^2+^ and BPQDs, resulting in an increased ECL signal. BPQDs and MXenes exhibit good photothermal properties, making them suitable for application as thermal converters in photothermal biosensors for exosome analysis. This study used a dual-modality probe of MXenes-BPQDs to create a biosensor. This approach not only expanded the use of MXenes and BPQDs in biodetection but also provided a reliable way for detecting exosomes [62].

In this research, Pang et al. created a self-powered biosensing device using titanium dioxide (TiO_2_) nanosilks (NSs) and molybdenum disulfide (MoS_2_) quantum dots (QDs). The device is used to detect exosomal RNA Homo sapiens HOXA distal transcript antisense RNA, (HOTTIP). This self-powered device provides higher power production than TiO_2_ NSs alone. The hybridization of TiO_2_ NSs with MoS_2_ QDs results in a heterojunction structure with an appropriate band offset, known as straddling (Type I) band alignment. MoS_2_ QDs’ sensitization and visible light absorption extend carrier lifespan and improve energy conversion efficiency. This self-powered biosensing gadget successfully detects HOTTIP quantitatively by hybridizing a capture probe with HOTTIP. HOTTIP capture results in a decrease in power output, allowing for ultrasensitive quantitative detection at concentrations ranging from 5 fg/mL to 50,000 ng/mL. The LOD was as low as 5 fg/mL. The TiO_2_ NSs@MoS_2_ QD-based nanomaterial has great potential for self-powered biosensing devices that are both cost-effective and portable. This self-powered, visible-light-driven gadget has promising applications for cancer biomarker quantitative detection [63].

**III. Natural Nanomaterials**: A whole new idea of physical mass amplification based on programmable molecular machinery is introduced by natural nanomaterials, particularly DNA-based nanostructures. These systems use autonomous molecular motion, strand displacement, rolling-circle amplification, and walker-based cascades to amplify signals rather than relying solely on inherent material features.

Precise stoichiometric control, logical signal processing, and dynamic amplification are made possible by DNA nanoparticles, which surpass the capabilities of static materials. They are excellent at converting a single recognition event into hundreds or thousands of downstream reporter molecules in exosome biosensing, thereby converting nanoscale vesicle binding into signals that can be measured macroscopically.

Crucially, natural nanomaterials have unparalleled selectivity and versatility, making them ideal for detecting subtle chemical differences among exosome subpopulations, a prerequisite for precision diagnostics.

(i) DNA Nanomaterials: As DNA nanoparticles allow for programmable, enzyme-free signal amplification through independent molecular motion and strand displacement, they are particularly significant. DNA nanomaterial systems represent molecular or information-based signal amplification rather than physical mass amplification.

Cao et al. developed a “principle-of-proof” biosensing approach for detecting exosomes using an immortal cell line (HepG2)-derived models. Target exosomes are concentrated on anti-CD63-functionalized immunobeads and recognized by a DNA chain with a CD63 aptamer region. This activates a catalytic molecular machine that uses a cascade-mediated strand displacement process. The molecule machine’s high efficiency allows for a linear range of 1 × 10^5^ to 5 × 10^7^ particles/mL and an LOD of 1.72 × 10^4^ particles/mL for exosomes, outperforming other approaches. The method’s excellent specificity in serum samples implies its potential utility in clinics for illness diagnosis, including early detection and prognosis monitoring of cancers [64].

**Figure 12:**
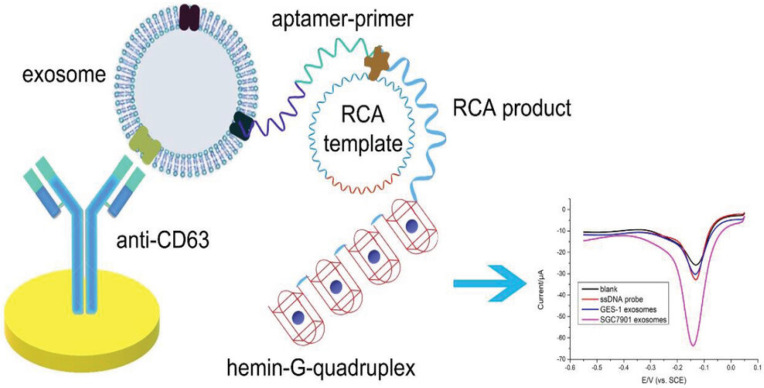
Illustration of the label-free electrochemical aptasensor for highly sensitive detection of exosomes [65].

Huang et al. demonstrated a label-free electrochemical aptasensor for detecting stomach cancer exosomes. This platform includes an anti-CD63 antibody-modified gold electrode and a gastric cancer exosome-specific aptamer. The aptamer is coupled to a primer sequence that matches a G-quadruplex circular template. Target exosomes can cause rolling circle amplification, resulting in numerous G-quadruplex units. This horseradish peroxidase-imitating DNA enzyme may reduce H_2_O_2_ and generate electrochemical signals. This aptasensor has remarkable selectivity and sensitivity for gastric cancer exosomes, with an LOD of 9.54 × 10^2^ mL^−1^ and a linear response range of 4.8 × 10^3^ to 4.8 × 10^6^ exosomes/mL. The electrochemical aptasensor could aid in the early detection of stomach cancer [65]. Figure 12 depicts the illustration of the label-free electrochemical aptasensor for highly sensitive detection of exosomes.

Dong et al. developed an aptamer-based method for detecting exosomes that involves releasing multiple DNAs and cyclic enzymatic amplification. The authors used aptamer-magnetic bead bioconjugates to capture tumor exosomes from LNCaP cells, resulting in the release of three types of messenger DNAs (mDNAs). Following magnetic separation, liberated mDNAs hybridized with probe DNAs fixed on a gold electrode. The signal reporter, electroactive Ru(NH_3_)_6_^3+^, was chosen for its electrostatic affinity to DNA. The electrochemical signal “turned off” after Exo III cyclic digestion. The electrochemical signal reflects the concentration of Ru(NH_3_)_6_^3+^, which is correlated with the concentration of mDNA, which is correlated with the concentration of exosomes. Therefore, tumor exosomes can be detected by examining the decrease in the peak current of Ru(NH_3_)_6_^3+^. This work found that the signal was increased by mDNA released from the magnetic bead and Exo III-assisted recycling. The best settings resulted in an LOD of 70 particles/μL, which is lower than most current approaches. This technique can detect tumor exosomes in complicated biological materials, indicating its potential for real-world diagnostics [66].

Zhang et al. created a ratiometric electrochemical biosensor using bipedal DNA walkers to detect exosomal MicroRNA 21 (miR-21) at an attomolar level. MiR-21 activates DNA walkers, leading to conformational modifications and increased signal ratios for both target-responding and target-independent reporters. The biosensor’s signal cascade amplification of DNA walkers results in super high sensitivity, with an LOD of 67 aM. The biosensor can detect exosomal miR-21 from breast cancer cell lines and serums due to the background correction function of reference reporters. The biosensor is highly selective, even against single-base mismatched targets, due to the exceptional discriminative capabilities of LNA-modified TMSDR. This sensor is regenerative and can withstand at least 5 cycles without losing sensitivity. The suggested biosensor’s high sensitivity, selectivity, and repeatability, combined with its low cost, make it a potential technique for detecting exosomal miRNAs in conjunction with early POCT for cancer [67].

Zhao et al. found that recognizing exosomes using aptamers resulted in the detection of DNA. CD63 and EpCAM aptamers were utilized to detect MCF-7 cell-secreted exosomes. The recognition technique was enhanced using a three-dimensional DNA walker. An Exonuclease III-assisted electrochemical ratiometric sensor was used for additional signal amplification. Under ideal conditions, the LOD of 1.3 × 10^4^ particles/mL was achieved with high selectivity. The clinical application test for detecting exosomes in human serum was also validated [68].

In order to achieve the precise and non-destructive separation of exosomes from intricate biological environments, Tang et al. devised a DNA-based hydrogel. The isolated exosomes were used in both the treatment of myocardial infarction in rat models and the direct detection of human breast cancer in clinical samples. This approach’s materials chemistry foundation included complementary base pairing to create DNA hydrogels and enzymatic amplification to create ultralong DNA chains. The ability of these ultralong DNA chains containing polyvalent aptamers to identify and interact with the receptors on exosomes facilitated the selective separation of exosomes from media into the subsequently created networked DNA hydrogel. This DNA hydrogel served as the basis for introducing logically constructed optical modules for the detection of exosomal pathogenic microRNA, resulting in 100% accurate categorization of breast cancer patients vs. healthy donors. Additionally, it was demonstrated that the DNA hydrogel containing exosomes produced from mesenchymal stem cells had considerable therapeutic effectiveness in healing the infarcted heart of rat models. The biological separation method based on DNA hydrogel has great promise as a potent biotechnology that will advance the creation of extracellular vesicles in nanobiomedicine [69].

He et al. developed an ultrasensitive method to detect and quantify tumor Exos in plasma microsamples (1 μL) at the single-vesicle level. The assay involves attaching activatable aptamer probes (AAP) to Exos collected by Exospecific antibodies on the surface of a flow cell, resulting in activated fluorescence. Furthermore, the attached AAP causes in situ assembly of a DNA nanodevice with improved fluorescence, improving Exo-detection sensitivity. A TIRF test for PTK7-Exo identifies tyrosine-protein-kinase-like 7 (PTK7) and separates target tumors from controls. This assay helps track tumor development and early treatment responses. The new test is adaptable for detecting and monitoring various disease-related Exo biomarkers [70].

**IV. Polymers**: Due to their three-dimensional structure, mechanical flexibility, and stimulus-responsive activity, polymer nanoparticles offer a structurally flexible foundation for mass magnification. In addition to providing active capture, release, and signal modulation in response to external stimuli like electrical fields or chemical triggers, conducting polymers, nanowires, and polymeric scaffolds can significantly enhance effective surface area.

Polymer nanostructures often function as integrated capture–amplification matrices in exosome sensing, where embedded conductive or redox-active domains transduce binding events into amplified signals, and nanoscale topography improves vesicle trapping. They are also ideal for point-of-care devices and scalable manufacturing due to their mechanical flexibility and processability.

(i) Polypyrrole Nanotubes: Through their three-dimensional structure and stimulus-responsive activity, polypyrrole nanotubes offer a mechanically and electrically active framework that improves mass magnification. Reusable, inexpensive sensing systems with high capture efficiency are enabled by their ability to reversibly capture and release exosomes in response to chemical or electrical stimuli.

**Figure 13:**
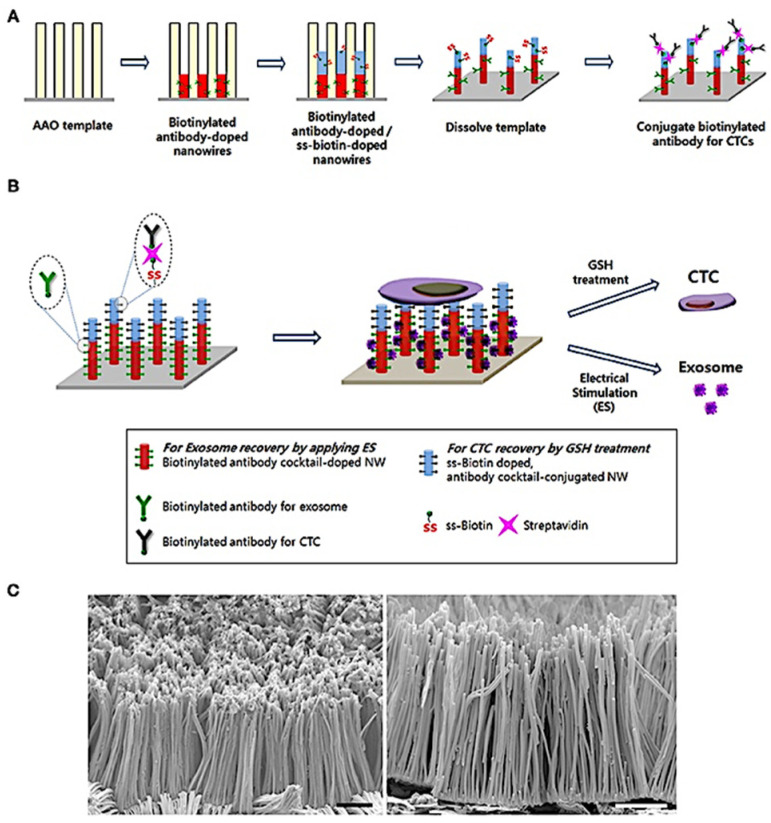
(**A**) Schematic of the process to synthesize the Ppy nanowire (NW) platform conjugated with simultaneously exosome-specific antibodies and CTC-targeting antibodies. (**B**) Schematic illustration of exosome and circulating tumor cells (CTCs) recovery from the same NW array (200 nm in diameter and 14 μm in length). This newly introduced platform possesses unique features by means of (i) doping exosome-specific biotinylated antibodies (i.e., anti-CD9, anti-CD81, and anti-CD63) to the bottom of the NW that could greatly enhance exosome isolation via the application of electrical stimulation (ES) and (ii) doping disulfide (SS)-biotin to the top of the NW that is further conjugated with CTC-targeting antibodies (i.e., anti-EpCAM, anti-EGFR, anti-vimentin, anti-N-cadherin, and anti-Trop2), which consequently releases the captured CTCs from the NW platform via GSH treatment. (**C**) Scanning electron microscopy images of the nanowire platform (scale bars = 5 μm) [71].

Lim et al. developed a simple electrochemical approach to efficiently catch and release exosomes and circulating tumor cells (CTCs) in a single platform with a well-ordered 3D architecture. Polypyrrole nanowires (Ppy NWs) are coupled with monoclonal antibodies that target marker proteins on exosomes or CTCs. The NW platform allows for precise retrieval of trapped exosomes or cells through electrical or glutathione (GSH) activation. Nano-topographic surfaces may recognize and trap small exosome-like vesicles (30–100 nm) through topographical interactions, while inhibiting bigger vesicles (100–1000 nm). Vertically aligned features boost cell capture efficiency when modified with high-affinity proteins. Exosomes and CTCs can be isolated from cancer patients’ blood samples using a single NW platform by modulating electrochemical and chemical cues. This approach has great potential for cancer diagnosis and downstream analysis due to its ease of use, effectiveness, and low cost [71]. Figure 13 presents a schematic of the process to synthesize the Ppy nanowire (NW) platform conjugated with simultaneously exosome-specific antibodies and CTC-targeting antibodies.

### 3.2. Cross-Class Comparison: Strengths, Limitations, and Design Trade-Offs

Comparative analysis, as opposed to paper-by-paper analysis, reveals some cross-cutting design trends despite the significant differences in composition and transduction method across the nanomaterial classes outlined above. First, the most practically usable platform is not always linked to the lowest nominal detection limit. Extremely low limits of detection are often reported by DNA walkers, RCA-based systems, ECL-active probes, and some plasmonically amplified assays; however, these values are often obtained under ideal laboratory conditions and may rely on multistep workflows, limited buffer windows, or extensive signal processing. In contrast, because they better handle analyte recovery, matrix interference, and assay reproducibility, platforms based on magnetic enrichment, metal nanoparticles, or robust hybrid nanocomposites frequently perform more reliably in real biofluids, despite reporting somewhat lower analytical sensitivity.

Second, various analytical bottlenecks are solved by the evaluated materials, and this differentiation provides more information than material identity alone. When inadequate physical loading is the main issue, dense metallic nanoparticles, magnetic nanocomposites, and certain polymer-supported topologies are more pertinent, particularly for mass-sensitive or resonant biosensors. On the other hand, where poor optical or electrochemical transduction, rather than low attachment mass, is the main factor limiting exosome detection, plasmonic, catalytic, fluorescent, and nucleic acid-mediated methods are more suitable. Two-dimensional and carbon-based nanomaterials frequently occupy an intermediate position: they are very appealing for interfacial signal propagation and miniaturized electronics, but surface fouling, oxidation, defect-state variability, or batch-to-batch inconsistency may have a greater impact on their performance.

Third, real-world usability and proof-of-concept sensitivity do not scale uniformly across material classes, which presents a persistent translational issue. Only a small percentage of studies show strong function in blood, plasma, urine, or patient-derived samples, despite the fact that many claim great analytical performance in buffer or reduced matrices. Because both exosome capture and signal production can be impacted by numerous proteins, lipoproteins, nonspecific adsorption, ionic-strength fluctuation, and colloidal instability, matrix effects continue to be a major barrier. Because they simultaneously increase enrichment, lower background, and allow for more reliable analytical output in complicated matrices, some self-calibrated hybrid designs seem particularly promising in this regard. Magnetic nanoparticles are highly useful for exosome enrichment in many assay formats, but they are not universally optimal for all mass-sensitive biosensors. In magnetoelastic and related field-responsive resonant systems, stable non-magnetic mass-loading strategies may be preferable. Although enrichment-assisted systems are frequently promising in complex biofluids, freely mobile magnetic nanoparticles may not always be preferred for magnetically actuated or field-biased resonant transducers due to their potential to disrupt resonance behavior, interfacial stability, or field distribution. Non-magnetic dense labeling or immobilized heavy-mass amplification techniques would be more appropriate in certain situations.

Fourth, there are still a number of common technical obstacles in the sector that go beyond particular classes of nanomaterials. Exosome heterogeneity, a lack of universal marker panels, insufficient pre-analytical handling uniformity, inconsistent recovery and matrix effect reporting, and poor interlaboratory repeatability are some of these. Furthermore, some of the most delicate systems depend on intricate multicomponent assemblies that could be challenging to produce consistently or use in standard clinical settings. Therefore, from a translational standpoint, the most important question is not just which nanomaterial produces the lowest LOD but also which one offers the greatest balance between recovery, selectivity, stability, ease of workflow, affordability, and compatibility with the targeted sensing platform.

When taken as a whole, these comparisons suggest that the choice of nanomaterials should be based on application rather than material. Increasing effective and mechanically stable physical loading at the sensor interface without sacrificing resonance behavior or field control is the most beneficial approach for researchers working on mass-sensitive MEMS, acoustic, cantilever, or magnetoelastic transducers. Because magnetically sensitive labels may become unwanted in field-biased or magnetically actuated systems due to unintentional motion, redistribution, aggregation, or modification of the transducer response, magnetic nanoparticles are not always the best materials in this situation. Immobilized heavy-mass labels, structurally stable hybrid composites, or dense yet magnetically passive nanoparticles would be better appropriate for such platforms. In contrast, non-gravimetric signal amplification could be more advantageous for integrated electrochemical, optical, or hybrid biosensors. Therefore, logical platform-compatible amplification techniques that align with the physics of the transducer, the biological matrix, and the planned diagnostic process are the most promising future path rather than a single universal material.

This comparative perspective provides the basis for the classification and translational analysis developed in Section 3, where the reviewed nanomaterial platforms are re-evaluated according to amplification mechanism, practical workflow value, and suitability for clinically relevant exosome sensing. Table 1 represents synthesis and characterization considerations for nanomaterials used in exosome sensing. Detailed synthetic processes for each nanomaterial are not replicated because this is a review rather than an experimental methodology study. However, material synthesis, surface functionalization, and characterization significantly impact the repeatability of nanomaterial-assisted exosome sensors. As a result, Table 1 offers a succinct overview of typical synthesis pathways, characterization techniques, and repeatability issues.

## 4. Comparison Between Different Particle Types and Their Review

Primarily relying on a single amplification method, nanomaterial-assisted mass magnification for exosome isolation and sensing results from a material-dependent link between biological recognition and physical signal transmission. Nanomaterials can be assessed based on (i) dominant amplification modality, (ii) effective mass or signal multiplication capacity, (iii) integration with sensing platforms, and (iv) clinical translatability limits, according to the studies described in Section 2. This comparative study provides a logical framework for selecting nanomaterials suited to specific liquid biopsy requirements.

### 4.1. Notes on Comparing Analytical Performance Across Studies

Since studies vary in target definition, calibration material, sample matrix, separation technique, and readout mode, detection limits reported in the exosome biosensing literature are not directly interchangeable. While some assays provide exosomal protein concentration, exosomal nucleic acid concentration, target-marker abundance, or similar values obtained from spiking standards, others report total vesicle concentration as particles/mL or particles/µL. Furthermore, because matrix proteins, lipoproteins, salts, viscosity, and nonspecific adsorption can significantly alter capture efficiency and background signal, detection limits measured in purified buffer or cell culture medium cannot be directly compared with those obtained in serum, plasma, urine, or patient samples.

Therefore, the detection limits summarized in this review are presented as representative values within their original assay context rather than as normalized rankings across material classes. Where possible, the tables now indicate the analyte definition, sample matrix, and whether the assay was demonstrated in buffer, spiked biological fluids, cell-culture medium, or clinical samples. Claims of superior performance are made only when the compared assays use similar target definitions and sample matrices.

### 4.2. Classification by Dominant Amplification Mechanism

Nanomaterial-assisted exosome sensing should be classified according to the dominant amplification mechanism rather than material composition alone. In this review, three functional categories are used.

First, predominantly physical mass-loading materials refer to dense labels, particle aggregates, or immobilized nanocomposites that increase the effective gravimetric or inertial load associated with exosome capture. These strategies are most relevant to QCM, SAW, microcantilever, magnetoelastic, and other resonant or mass-sensitive transducers. Dense metallic nanoparticles, heavy metal-compound nanoparticles, and stable polymer–nanoparticle composites can fall into this category when their primary function is to increase the mass coupled to the sensor interface.

Second, predominantly non-gravimetric signal-amplifying materials refer to nanomaterials whose main contribution is optical, electrochemical, catalytic, fluorescent, chemiluminescent, or nucleic-acid-mediated signal gain rather than physical mass increase. Carbon nanomaterials, MXenes, quantum dots, upconversion nanoparticles, DNA walkers, rolling-circle amplification systems, and many ECL nanoprobes belong mainly to this category. These materials may have physical mass, but their analytical advantage usually arises from enhanced charge transfer, luminescence, catalytic turnover, plasmonic coupling, or reporter multiplication.

Third, hybrid enrichment/mass-loading/signal-amplifying systems combine more than one function, such as magnetic enrichment followed by electrochemical readout, MOF-based reporter loading, AuNP-assisted plasmonic enhancement, or dense nanocomposite-assisted mass and catalytic amplification. These systems are common in exosome analysis because enrichment, matrix cleanup, and signal generation are often integrated in a single workflow.

This mechanism-based classification avoids treating all nanomaterials as “mass amplifiers” and clarifies whether the amplified quantity is physical mass, optical/electrical signal, reporter number, or local exosome concentration. Table 2 describes the mechanism-based classification of nanomaterial-assisted exosome isolation and sensing strategies.

### 4.3. Amplification Physics Across Nanomaterial Classes

The physical realization of amplification is a crucial difference across types of nanomaterials:

Because of their high atomic density, plasmonic coupling, and catalytic activity, metallic and metal compound nanoparticles are ideal for optoelectronic and mass-sensitive sensors. While CuS, CuO, and hybrid metal oxides provide chemiluminescent, enzyme-free catalytic amplification, gold and silver nanoparticles are excellent for aggregation-based colorimetric tests and plasmon-enhanced fluorescence [19,21,23,24,25,26,28,29,40,72].

By actively concentrating exosomes at the sensor interface, magnetic nanoparticles overcome diffusion-limited transport and make a unique contribution to mass magnification. They play a dual role in improving signal-to-noise ratios in complex biofluids by facilitating matrix purification and boosting local analyte density [25,30,31].

Structural amplification is enabled by MOFs and zirconium-based coordination systems, which allow dense loading of electroactive or fluorescent reporters while maintaining molecular specificity through ultrahigh porosity and coordination chemistry. Self-calibrated and label-free detection techniques with lower assay variability are supported by these systems [33,34,49].

Through conductive networks and catalytic mimics, carbon-based and two-dimensional nanomaterials enhance signals, enabling binding-induced perturbations to spread over wide interfacial regions. ECL and electrochemical platforms demonstrate their efficacy by outperforming traditional ELISA by two to three orders of magnitude [51,52,54,55,56].

DNA nanomaterials represent a distinct non-gravimetric amplification paradigm in which signal gain arises from stoichiometric reporter multiplication and programmable molecular reactions rather than physical mass increase. Rolling-circle amplification, DNA walkers, and strand-displacement cascades can convert a single exosome-recognition event into multiple optical or electrochemical outputs. However, the extremely low detection limits reported for these systems should be interpreted in the context of the original assay conditions. Many values are obtained in buffer, cell-culture medium, or spiked serum/plasma, whereas fewer studies have been validated in large independent clinical cohorts. Therefore, these methods demonstrate excellent analytical potential, but their clinical robustness depends on nuclease stability, matrix interference, sample preparation, and validation in real patient samples. [64,65,66,67,68,70].

Polymer nanostructures, especially conducting polymers like polypyrrole, combine electrical conductivity, stimulus-responsive release, and nanoscale topography to function as integrated capture–amplification matrices. These characteristics are particularly appealing for point-of-care and reusable platforms [71]. Table 3 summarizes the dominant amplification mechanisms across nanomaterial classes for exosome isolation and sensing.

### 4.4. Translational Considerations and Platform Compatibility

The ongoing application of ultracentrifugation as the standard comparator for exosome extraction is a crucial translational factor in this research. Although differential ultracentrifugation has long been one of the most popular methods for studying extracellular vesicles, its practical drawbacks are increasingly acknowledged. These include labor-intensive multistep methods, lengthy processing periods, high capital and operating costs, comparatively low recovery in some sample types, and the frequent co-enrichment of non-vesicular pollutants, including lipoproteins and protein complexes. Large centrifugal forces may also promote vesicle aggregation or alter vesicle integrity, potentially jeopardizing downstream quantitative analysis. Therefore, it is important to consider whether new nanomaterial-assisted techniques improve clinically relevant performance metrics such as recovery, purity, throughput, sample-volume efficiency, and compatibility with integrated sensing, in addition to whether they replicate ultracentrifugation-based workflows. According to this viewpoint, magnetic, plasmonic, and hybrid nanostructured platforms present a significant opportunity to transition from workflows focused on ultracentrifugation to more application-specific and diagnostically useful exosome-processing techniques.

Although hybrid mass-loading/signal-amplifying system techniques often yield the lowest detection limits, scaling may be hindered because they often require sophisticated surface chemistry, multistep fabrication, or external-field modification. On the other hand, signal-amplifying nanomaterial platforms, especially those based on AuNPs and MOFs, offer a good compromise between manufacturability, robustness, and sensitivity.

The analytical performance of small-mass-magnification techniques, particularly those based on DNA nanotechnology and ECL-active nanoparticles, is excellent; however, for clinical use, nucleic acid stability and assay conditions must be carefully controlled. Because of their inherent capacity for enrichment, magnetic-assisted systems routinely perform better in actual biofluids, making them extremely compatible with regulatory-grade workflows.

Importantly, matrix-dependent interference from serum proteins, lipoproteins, and nonspecific adsorption frequently modifies test recovery and signal quality; translational evaluation cannot rely just on the detection limit. To illustrate the difference between proof-of-concept buffer performance and validation in clinically relevant samples such as plasma, serum, urine, and protein-rich media, Table 1, Table 2 and Table 3 have been expanded to include representative real-sample recovery and matrix compatibility metrics from the referenced studies.

Analytical performance alone does not establish clinical diagnostic usefulness, despite the remarkable analytical sensitivity of several nanomaterial-assisted exosome tests. Clinical translation requires validation in sufficiently powered and independent patient cohorts, standardized pre-analytical handling, robust EV characterization, interference testing against lipoproteins and protein aggregates, reproducible recovery, and comparison with established clinical endpoints. Many of the current investigations are still proof-of-concept experiments that use tiny serum/plasma cohorts, spiked samples, or exosomes produced from cell lines. As a result, rather than being presented as clinically proven, the clinical claims in this evaluation are intentionally framed as translational potential.

Overall, the literature examined indicates that no single nanomaterial is ideal in every situation. Rather, the transduction mode, target biomarker, sample matrix, and intended clinical use must all be considered when selecting a mass magnification agent. Table 4 describes translational considerations and platform compatibility of nanomaterial-based mass magnification strategies for exosome sensing.

### 4.5. Clinically Oriented Case Examples and Translational Status

Most nanomaterial-assisted exosome sensors remain in the proof-of-concept or early translational stages rather than being routinely used in clinical settings, despite several having been tested in patient-derived samples. Nonetheless, several therapeutically focused examples demonstrate how nanomaterial platforms could aid the development of liquid biopsies. Due to their straightforward optical readout and minimal equipment requirements, gold nanoparticle-based colorimetric and plasmonic assays have been used for exosome surface marker profiling. To improve matrix cleanliness and local target concentration before electrochemical or optical detection, magnetic nanoparticle-assisted systems have been employed to enrich exosomes from serum or plasma. Ratiometric systems based on MOFs and black phosphorus have enabled self-calibrated exosome detection in serum/plasma samples, thereby reducing matrix-related signal variability. Serum small extracellular vesicle profiling in prostate cancer-related applications has also been investigated using TiN/Al plasmonic metasurface platforms.

Although there is translational potential in these cases, they should not be considered established clinical diagnoses. Before clinical adoption, most studies still need larger independent cohorts, blinded validation, consistent pre-analytical handling, robust EV characterization, and comparability with established clinical outcomes. Table 5 depicts clinically oriented examples of nanomaterial-assisted exosome sensing.

## 5. Conclusions

The analytical toolkit for exosome and extracellular vesicle separation and sensing has significantly increased thanks to nanomaterial-assisted techniques. The capacity to tailor material characteristics to the needs of particular transducers, biomarkers, sample matrices, and clinical use cases is what gives them their value rather than a single universal amplification mechanism. Dense nanomaterials can increase the effective mass associated with the sensor surface for gravimetric and resonant sensors. On the other hand, non-gravimetric mechanisms like enhanced charge transfer, reporter loading, catalytic turnover, plasmonic coupling, fluorescence modulation, or molecular signal multiplication are more frequently advantageous for optical, electrochemical, fluorescent, catalytic, plasmonic, and nucleic acid-based platforms. As a result, differentiating between physical mass loading and non-gravimetric signal amplification provides a realistic tool for choosing amplification techniques based on sensing physics rather than a universal framework.

Many nanomaterial-assisted exosome assays remain in the proof-of-concept or early translational stages, despite their remarkable analytical sensitivity. Low detection limits will not be enough for clinical acceptance. Standardized pre-analytical handling, repeatable isolation and recovery, trustworthy purity evaluation, robust EV characterization, interference testing against lipoproteins and protein aggregates, scalable fabrication, batch-to-batch reproducibility, inter-laboratory validation, and regulatory-compatible performance evaluation are among the essential requirements. Because exosome content, cargo composition, and surface-marker expression can change depending on disease state, tissue origin, treatment status, sample type, and patient-to-patient variation, biological variability presents another significant hurdle. These problems underscore the necessity of multiplexed marker panels, internal controls, and validation in separate clinical cohorts with adequate power. 

Magnetic nanoparticles are highly valuable for exosome enrichment, preprocessing, and matrix cleanup. However, their suitability is platform-dependent. In magnetically biased or field-actuated resonant sensors, including magnetoelastic platforms, freely mobile magnetic labels may perturb field distribution, interfacial stability, or resonance behavior. For these architectures, immobilized or magnetically passive dense labels may provide a more controllable route to physical mass loading.

Physical mass loading may be especially significant for mass-sensitive platforms, such as magnetoelastic, acoustic, QCM, and cantilever-based sensors. However, they only cover a small portion of the larger region of exosome sensing. Therefore, rather than assuming that a single nanomaterial class, amplification mechanism, or sensing modality is generally ideal, future advancements should focus on application-specific integration of isolation, amplification, and transduction.

### 5.1. Current Challenges in Translational Exosome Mass Magnification Platforms

Despite these developments, there are still several unresolved translational obstacles. First, there is still significant platform dependence in long-term material and signal stability. As an illustration of the potential for strong storage stability, the Au nanoparticle–MoS_2_–rGO nanocluster-doped SAW biosensor reported by Jandas et al. maintained significant activity with only ~10% loss over 80 days [73]. However, oxidation, hydrolysis, surface reconstruction, or signal drift in complex biofluids may make many highly sensitive systems based on 2D materials, hybrid thin films, or porous coordination networks more susceptible, especially after extended storage or recurrent use [13,33,34,54,62]. This implies that shelf-life, operational stability, regeneration efficacy, and calibration drift under clinically relevant storage settings should be more methodically reported in future research. Limitations may be lessened by techniques like core–shell structures, internal self-referencing designs, and antifouling/passivating coatings.

Second, batch-to-batch reproducibility and inter-laboratory robustness remain lacking, despite several studies reporting encouraging analytical repeatability. Examples include >90% recovery utilizing anion-exchange magnetic beads for plasma exosome separation [36] and %RSD values < 5.5% in ferric oxide nanozyme-assisted exosome assays and quantum dot/electrochemical platforms [25,57,59]. However, these positive findings are often obtained in very small validation cohorts under optimal laboratory conditions. Future investigations should more strictly standardize nanoparticle production, ligand density, colloidal size distribution, storage conditions, and inter-assay/inter-operator variability for clinical translation. This should preferably be done in conjunction with benchmarking against recognized EV characterization techniques.

Third, one of the primary obstacles remains uniformity across biological matrices. The practical potential of nanomaterial-assisted tests in therapeutically relevant settings has been confirmed by several methods described here, which have demonstrated feasibility in blood, plasma, urine, cell culture medium, or 10% FBS [26,29,34,49]. However, capture efficiency and transduction fidelity can be significantly affected by matrix-dependent interference from numerous proteins, lipoproteins, nonspecific adsorption, changes in ionic strength, and protein corona formation. Because many stated detection limits are determined in buffered systems or partially controlled samples rather than in truly diverse clinical matrices, this is especially crucial. Antifouling surface engineering, pre-analytical harmonization, reference EV materials, and consistent reporting requirements for sample preparation, normalization, and analytical recovery should thus be given more attention in future studies.

### 5.2. Future Outlook: Hybrid Multimodal and AI-Assisted Design Strategies

The creation of hybrid multimodal systems that integrate orthogonal amplification techniques into a single process is a particularly attractive avenue. This direction has already been indicated by several studies, such as magnetic enrichment combined with optical or electrochemical readout [25,31], self-calibrated hybrid thin films incorporating MOFs and black phosphorus nanosheets [34], and MXene-based electrochemiluminescent architectures further enhanced with in situ formed Au nanoparticles [13,54]. By concurrently addressing target enrichment, signal production, and internal correction, such multimodal systems can increase resilience, lower false positives, and enhance performance in complicated matrices. In general, technologies that include multiplexed electrochemical or optical outputs, nanostructured capture interfaces, magnetic preconcentration, and microfluidics are expected to provide the optimal balance among clinical usability, workflow simplicity, and sensitivity.

Adoption of multiplexed and self-calibrated sensor designs is another significant future path. Single-marker tests may be inadequate for accurate disease stratification due to the inherent heterogeneity of exosome populations. While multiplexed detection of protein and nucleic acid cargos may better capture disease-specific exosome markers, dual-signal or ratiometric outputs, as previously shown in some MOF-hybrid systems [74], might enhance assay accuracy.

Lastly, the logical development of next-generation exosome sensing systems may be accelerated by AI-assisted design. In addition to enhancing signal processing and multimarker classification from electrochemical, plasmonic, fluorescence, or ECL datasets, machine learning may help optimize nanoparticle size, composition, shape, ligand density, and antifouling chemistry. AI-guided materials discovery and assay optimization are still in their infancy. Still, they have the potential to reduce development cycles, increase repeatability, and facilitate more accurate interpretation of diverse clinical data.

In conclusion, enhancing stability, repeatability, standardization, and integration into therapeutically feasible processes will be just as important to the future of nanomaterial-assisted exosome sensing as lowering detection limits. Together, hybrid multimodal architectures, self-calibrated sensing methods, and AI-assisted design methodologies offer a reliable path from proof-of-concept demonstrations to reliable future precision diagnostic platforms for exosome mass magnification platforms.

Most importantly, future developments in exosome analytics should prioritize fit-for-purpose isolation and sensing techniques that maximize recovery, purity, speed, and clinical deployability, rather than relying on ultracentrifugation as the standard.

### 5.3. Future Outlook: Evidence-Based Directions for Clinically Relevant Exosome Sensing

Rather than solely reducing analytical detection limits, future advancements in nanomaterial-assisted exosome sensing should focus on enhancing clinical reliability. Standardized reporting of sample preparation, isolation recovery, purity, EV characterization, and matrix effects should be the top emphasis. Future research should use orthogonal characterization techniques and appropriate negative controls to ensure that the measured signal originates from the intended vesicle population, as exosomes and small extracellular vesicles physically and biochemically overlap with lipoproteins, protein aggregates, and other extracellular particles.

Enhanced performance in therapeutically important biofluids is a second priority. Although serum, plasma, urine, saliva, and other biofluids include nonspecific adsorption, protein corona formation, ionic-strength change, nuclease activity, and optical or electrochemical background, many nanomaterial-assisted sensors have high sensitivity in buffer or spiked samples. Therefore, ratiometric readouts, internal standards, antifouling surface chemistries, and self-calibrated sensor designs are probably more crucial for translation than merely slight increases in detection limit.

Multiplexed and orthogonally verified detection is a third direction. Because marker expression varies across cell types, disease stages, and patient populations, single exosomal markers such as CD63, CD9, CD81, EpCAM, HER2, or EGFR may not be sufficient for disease categorization. When paired with independent validation techniques, multiplexed protein and nucleic acid panels may more effectively handle biological heterogeneity and lower false-positive or false-negative outcomes.

Lastly, scalable production, reagent stability, batch-to-batch repeatability, user-friendly procedures, and validation in appropriately powered independent cohorts will all be necessary for clinical translation. Nucleic acid amplification, mass-sensitive platforms, plasmonic and electrochemical readouts, magnetic or affinity-based enrichment, and microfluidic integration all have unique benefits, but none is always the best. Future systems that combine isolation, amplification, and detection in a manner appropriate for the intended clinical use case are probably going to be the most promising.

Multimarker categorization, signal processing, and material optimization may potentially be supported by AI-assisted design and data analysis. However, these methods should be used in conjunction with clinically representative sample testing, uniform reporting, and thorough experimental validation, not in place of them.

### 5.4. Research Gaps and Future Directions

For nanomaterial-assisted exosome detection to be developed into reliable liquid biopsy technologies, several research gaps need to be addressed. First, more consistency is needed for isolation and enrichment processes. Recovery, purity, vesicle size distribution, marker expression, and contamination by lipoproteins or protein aggregates should all be reported more reliably in future research. Second, matrix effects remain a significant obstacle. Proteins, salts, nucleases, and colloidal species found in serum, plasma, urine, and saliva can change the stability, surface binding, and optical or electrochemical backdrop of nanoparticles. Thus, matrix-matched calibration, ratiometric readouts, antifouling coatings, and internal standards should become standard.

Third, multiplexed detection is necessary due to exosome heterogeneity. Because vesicle subpopulations vary by tissue origin, disease stage, and patient background, single-marker tests might not be sufficient for disease classification. Nucleic acid and multiplexed protein panels are probably more therapeutically useful. Fourth, new systems need to be verified using separate, well-powered patient cohorts, preferably with blinded testing and comparison to recognized clinical objectives. Fifth, early platform development must account for manufacturing factors such as device-to-device variability, storage stability, ligand density control, and nanoparticle batch repeatability. Lastly, transducer physics should guide the selection of amplification techniques: programmable DNA amplification for molecular profiling, plasmonic or fluorescent labels for optical platforms, catalytic/electrochemical enhancers for electrode-based systems, and dense mass labels for resonant sensors. The shift from proof-of-concept exosome tests to useful liquid-biopsy technologies might be expedited by this platform-specific yet clinically based method.

## Figures and Tables

**Table 1:** Representative synthesis and characterization considerations for nanomaterials used in exosome sensing.

Nanomaterial Class	Representative Materials	Common Synthesis/Assembly Approaches	Key Characterization Methods	Reproducibility Considerations
Metallic nanoparticles	AuNPs, AgNPs, CuS, CuO	Citrate reduction, seed-mediated growth, solvothermal synthesis, surface ligand exchange	TEM/SEM, DLS, UV–vis, zeta potential, XPS, ICP-MS	Particle size, aggregation state, ligand density, batch-to-batch optical response
Magnetic nanoparticles	Fe_3_O_4_, Fe_3_O_4_@Au, magnetic graphene oxide	Co-precipitation, thermal decomposition, silica/gold coating, EDC/NHS or streptavidin–biotin conjugation	TEM/SEM, DLS, VSM, zeta potential, FTIR/XPS	Magnetic response, colloidal stability, nonspecific adsorption, antibody/aptamer loading
Carbon/2D materials	graphene, CNTs, g-C_3_N_4_, MXenes	Exfoliation, hydrothermal synthesis, etching/exfoliation for MXenes, surface functionalization	Raman, XRD, XPS, AFM, TEM, conductivity, zeta potential	Defect density, oxidation state, sheet size, surface fouling
Quantum dots/BPQDs	CdSe QDs, carbon dots, BPQDs	Colloidal synthesis, hydrothermal carbonization, liquid-phase exfoliation	Fluorescence spectra, quantum yield, TEM, XPS, DLS	Photostability, oxidation, toxicity, surface passivation
MOFs	Zr-MOFs, ZIF-8, Cu-BTC	Solvothermal synthesis, room-temperature coordination assembly, post-synthetic modification	XRD, BET, SEM/TEM, FTIR, XPS, electrochemical characterization	Pore stability, reporter loading, aqueous stability, batch reproducibility
DNA nanostructures	DNA walkers, RCA products, aptamer assemblies	Sequence-programmed hybridization, enzymatic amplification, strand displacement	Gel electrophoresis, fluorescence, melting analysis, PAGE, DLS when assembled on particles	Sequence purity, nuclease stability, enzyme activity, contamination control
Polymer nanostructures	polypyrrole nanowires, conductive polymer composites	Electropolymerization, template-assisted synthesis, surface bioconjugation	SEM, impedance, cyclic voltammetry, FTIR, contact angle	Film thickness, conductivity, aging, antifouling performance

**Table 2:** Mechanism-Based Classification of Nanomaterial-Assisted Exosome Isolation and Sensing Strategies.

Magnification Regime	Representative Nanomaterials	Main Amplified Quantity	Representative Sensing Platforms	Observations
Predominantly physical mass-loading	Dense AuNPs, metal-compound NPs, heavy nanocomposites, immobilized polymer–NP composites	Gravimetric/inertial load	QCM, SAW, cantilever, magnetoelastic, acoustic	Most relevant to mass-sensitive transducers
Predominantly non-gravimetric signal amplification	Carbon materials, MXenes, QDs, UCNPs, DNA walkers, RCA systems, ECL probes	Optical/electrical/catalytic/reporter signal	ECL, fluorescence, electrochemical, colorimetric	High sensitivity but not true physical mass amplification
Hybrid enrichment/signal/mass systems	Magnetic NPs, MOFs, AuNP/MOF hybrids, metal–semiconductor composites	Local concentration, reporter loading, catalytic/plasmonic output, sometimes mass	Magnetic–electrochemical, SPR, fluorescence, QCM/SAW hybrids	Useful in complex matrices but mechanism must be specified

**Table 3:** Amplification Physics Across Nanomaterial Classes for Exosome Isolation and Sensing.

Nanomaterial Class	Representative Materials	Primary Amplification Physics	Dominant Signal Contribution	Typical Sensing Modalities	Analytical Performance (Representative)	Representative Real-Sample Recovery/Matrix Effect	Key Strengths	Limitations & Translational Considerations
Metallic & metal compound nanoparticles	AuNPs, AgNPs, CuS, CuO, hybrid metal oxides	Plasmonic coupling, high atomic density, enzyme-free catalytic reactions	Optical enhancement, catalytic signal generation, physical mass loading	Colorimetric, plasmon-enhanced fluorescence, chemiluminescence, QCM	pM–fM equivalent exosome signals; rapid visual readout	Demonstrated in serum, plasma, urine, and conditioned media; some assays show good agreement without pre-isolation and direct plasma compatibility, but matrix-dependent interference can increase background	Strong optoelectronic response; facile synthesis; robust signal	Susceptible to aggregation artifacts; limited multiplexing; batch variability
Magnetic nanoparticles	Fe_3_O_4_, ferrites, magnetic nanocomposites	Active target enrichment and transport; diffusion barrier elimination	Local analyte concentration and effective mass magnification	Magnetic, electrochemical, QCM, hybrid assays	Near single-exosome sensitivity after enrichment	Particularly effective in complex matrices; plasma isolation with >90% recovery has been reported, with reduced protein impurity compared with ultracentrifugation	High signal-to-noise in complex biofluids; efficient matrix purification	Requires external magnetic control; increased system complexity
MOFs & coordination nanostructures	Zr-based MOFs, porphyritic MOFs, coordination polymers	Structural amplification via ultrahigh porosity and multivalent loading	Dense reporter packing with preserved molecular specificity	Electrochemical, fluorescence, ECL, label-free impedance	Sub-fM equivalent detection; low assay variability	Validated in serum/plasma and 10% FBS; self-calibrated designs help mitigate matrix variability, though long-term fluid stability remains important	Self-calibration potential; tunable chemistry; high reproducibility	Stability in aqueous biofluids; synthesis scalability
Carbon-based & 2D nanomaterials	Graphene, CNTs, MXenes, MoS_2_	Conductive percolation networks; catalytic mimicry; interfacial signal propagation	Electron-transfer amplification over extended interfaces	Electrochemical, ECL, FET-based sensors	10^2^–10^3^× sensitivity over ELISA	Successfully demonstrated in serum and patient-derived circulating exosome samples; however, surface fouling and defect-sensitive behavior can accentuate matrix effects	High sensitivity; rapid response; compatibility with miniaturization	Surface fouling; performance sensitive to defect density
DNA & natural nanomaterials	DNA nanostructures, aptamer assemblies, protein scaffolds	Stoichiometric and information-based amplification (RCA, DNA walkers, strand displacement)	Multiplicative reporter generation independent of mass	Fluorescence, ECL, electrochemical	Sub-100 exosomes; attomolar nucleic acid detection	Frequently validated in serum; strong analytical performance, but matrix compatibility depends on nuclease control and stringent assay conditions	Extreme sensitivity; high programmability; multiplexing capability	Long reaction times; enzymatic dependence; contamination risk
Polymer nanostructures	Polypyrrole, conductive polymer composites	Integrated capture–amplification matrices; stimulus-responsive signal modulation	Electrical conductivity and morphology-driven enhancement	Electrochemical, impedance, wearable/POC sensors	Low-pM to fM equivalent sensitivity	Real-sample compatibility promising, especially for integrated isolation platforms, but antifouling optimization remains necessary	Reusable platforms; mechanical flexibility; POC suitability	Long-term stability; material aging effects

**Table 4:** Translational Considerations and Platform Compatibility of Nanomaterial-Based Mass Magnification Strategies for Exosome Sensing.

Magnification Strategy/Nanomaterial Class	Typical Examples	Analytical Strength	Manufacturability & Scalability	Compatibility with Clinical Biofluids/Real-Sample Recovery	Platform Integration & Workflow Complexity	Regulatory & Translational Readiness	Representative Use Scenarios
Hybrid mass-loading/signal-amplifying systems platforms	Dense metallic NPs, magnetic nanocomposites, polymer–NP hybrids	Ultra-low detection limits; near single-exosome sensitivity	Moderate to low; multistep fabrication and complex surface chemistry often required	High when enrichment is integrated; selected plasma studies report recovery > 90%, though handling-dependent losses remain possible	High complexity; often requires external fields or multi-stage protocols	Moderate; performance strong but operational complexity is a barrier	High-sensitivity laboratory diagnostics; confirmatory assays
Signal-amplifying nanomaterials platforms	AuNPs, AgNPs, MOFs, metal oxides	High sensitivity with good reproducibility	High; scalable synthesis and established functionalization chemistries	Broadest validation in serum/plasma/urine; several systems directly tested in patient samples and protein-rich media such as 10% FBS	Moderate complexity; compatible with standard optical and electrochemical platforms	High; strong balance of performance and practicality	Routine liquid biopsy assays; translational research and early clinical testing
Physical mass-loading nanomaterials platforms	DNA nanostructures, ECL-active nanoparticles, QDs	Excellent analytical performance; very low theoretical LODs	Variable; DNA-based systems may require stringent handling	Moderate; often validated in serum, but recovery and robustness depend strongly on nuclease control and surface fouling management	Moderate to high; tight control of assay conditions required	Moderate; stability and robustness remain concerns	Ultra-sensitive research assays; molecular profiling
Magnetic-assisted hybrid platforms	Magnetic NPs + catalytic or electrochemical reporters	High sensitivity with improved SNR in real samples	Moderate; scalable but requires magnetic integration	Very high; strong matrix purification and enrichment effects, with some studies reporting >90% recovery and reduced protein impurity	Moderate; additional magnetic handling steps	Very high; aligned with regulatory-grade workflows	Clinical sample preprocessing; integrated isolation–detection
POC-oriented polymer nanostructures platforms	Conducting polymers (e.g., polypyrrole), flexible composites	Moderate to high sensitivity	High; compatible with printing and flexible electronics	Moderate; matrix effects manageable but antifouling and long-term drift remain relevant	Low to moderate; suitable for integrated devices	High for decentralized testing	Point-of-care diagnostics; reusable sensing platforms

**Table 5:** Clinically oriented examples of nanomaterial-assisted exosome sensing.

Platform/Material	Clinical Sample Context	Target/Readout	Translational Strength	Remaining Limitation	Clinical Sample Context
AuNP colorimetric/plasmonic assays	Serum/plasma or patient-derived biofluids in selected studies	Exosomal surface proteins	Simple visual/optical readout	Matrix interference, protein corona, cohort size	AuNP colorimetric/plasmonic assays
Magnetic nanoparticle enrichment	Plasma/serum/cell medium	CD63/EpCAM/HER2 or other markers	Enrichment and matrix cleanup	Recovery standardization, antibody bias	Magnetic nanoparticle enrichment
MOF/BP ratiometric aptasensor	Serum/plasma samples	Exosome markers/electrochemical dual signal	Self-calibrated output	Long-term stability, broader validation	MOF/BP ratiometric aptasensor
TiN/Al plasmonic metasurface	Serum sEV profiling	Plasmonic label-free detection	High-throughput optical sensing	Need independent clinical validation	TiN/Al plasmonic metasurface
DNA walker/RCA systems	Serum/spiked samples/small cohorts	Exosomal proteins or miRNAs	Very high analytical sensitivity	Nuclease stability, workflow complexity	DNA walker/RCA systems

## Data Availability

No new data were created or analyzed in this study. Data sharing is not applicable to this article.

## Abbreviations

The following abbreviations are used in this manuscript:

**Sr. No.**	**Abbreviations**	**Full Forms**
1.	SPR	surface plasmon resonance
2.	SAW	surface acoustic wave
3.	QCM	quartz crystal microbalance
4.	EVs	Extracellular vesicles
5.	CL	chemiluminescence
6.	HER2	Human epidermal growth factor receptor 2
7.	ELISA	Enzyme-linked immunosorbent assay
8.	CuNCs	copper nanoclusters
9.	HMSNs	hollow mesoporous silicon nanospheres
10.	AuNP	Gold nanoparticle
11.	TEXs	tumor-derived exosomes
12.	PLA	proximity ligation assay
13.	TMA	transcription-mediated amplification
14.	RPA	recombinase polymerase amplification
15.	NPC	nasopharyngeal carcinoma
16.	LPM1	latent membrane protein 1
17.	EGFR	epidermal growth factor receptor
18.	LOD	limit of detection
19.	PLAP	placenta alkaline phosphatase
20.	RSD	relative standard deviation
21.	LFIA	lateral flow immunoassay
22.	mulAu	multilayer gold nanoparticles
23.	monAu	monolayer gold nanoparticles
24.	NAM	nanoporous alumina membrane
25.	RCA	rolling circle amplification
26.	AgNPs	silver nanoparticles
27.	MEF	metal-enhanced fluorescence
28.	FITC’s	Fluorescein isothiocyanate’s
29.	PBS	phosphate-buffered saline
30.	sNEM	silver nanoparticle embedded membrane
31.	AChE	acetylcholinesterase
32.	n-SMase	neutral sphingomyelinase
33.	GC-SPR	grating-coupled surface plasmon resonance
34.	MGONs	magnetic graphene oxide nanoparticles
35.	GBM	Glioblastoma
36.	Zr-MOFs	Zr-based metal–organic frameworks
37.	BPNSs	black phosphorus nanosheets
38.	Fc	ferrocene
39.	ITO	indium tin oxide
40.	MB	methylene blue
41.	ZIF-8	Zeolitic imidazolate framework-8
42.	MNP/Cu-BTC MOF	magnetic nanoparticle-modified copper-based MOF
43.	SA-GOx	streptavidin–glucose oxidase
44.	AE	anion exchange
45.	UC	ultracentrifugation
46.	PCa	Prostate cancer
47.	ZnO	Zinc Oxide
48.	3D	three-dimensional
49.	PDMS	polydimethylsiloxane
50.	HRP	horseradish peroxidase
51.	TMB	3,3′,5,5′-tetramethylbenzidine
52.	MNP	magnetic nanoparticle
53.	EpCAM	epithelial cell adhesion molecule
54.	HCC	hepatocellular carcinoma
55.	MB	magnetic beads
56.	CuNPs	copper nanoparticles
57.	rGO@PDA	polydopamine-functionalized graphene oxide
58.	ATP	adenosine triphosphate
59.	BAF-TiN	biotinylated antibody-functionalized TiN
60.	AI	aluminum
61.	sEVs	small extracellular vesicles
62.	LRET	luminescence resonance energy transfer
63.	UCNPs	upconversion nanoparticles
64.	Au NRs	gold nanorods
65.	TAMRA	tetramethyl rhodamine
66.	ASPN	a near-infrared semiconducting polyelectrolyte
67.	ASPNC	afterglow signal of the nanocomplex
68.	L-TG-LRET	long-lived luminous UCNP donors
69.	miRNAs	microRNAs
70.	AuNPs@MCF/MWCNTs	Mesoporous carbon functionalized with gold nanoparticles
71.	POCT	Point-of-care testing
72.	ssDNA	single-stranded DNA
73.	NSs	nanosheets
74.	s-SWCNTs	Single-walled carbon nanotubes that being excellent water solubility
75.	GFET	graphene-based field-effect transistor
76.	SERS	surface-enhanced Raman scattering
77.	ECL	electrogenerated chemiluminescence
78.	MCF-7 cells	breast cancer cell line
79.	MCF-10 cells	breast cancer cell line
80.	AuNPs–MXenes–Apt	gold nanoparticles (AuNPs) and Ti_3_C_2_ MXenes hybrids with aptamer modification
81.	g-C_3_N_4_@Galinstan-PDA	g-C_3_N_4_ conjugated polydopamine-coated Galinstan liquid metal shell-core nanohybrids
82.	NPs	nanoparticles
83.	CdSeQD	CdSe quantum dot
84.	%RSD	relative standard deviation
85.	DSPE-PEG-Biotin	biotin-functionalized phosphatidylethanolamine
86.	MPA-CdS:Eu	Mercaptopropionic acid (MPA)-modified Eu3+-doped CdS nanocrystals
87.	NCs	nanocrystals
88.	BP	black phosphorus
89.	BPQDs	black phosphorus (BP) quantum dots (QD)
90.	Ru(dcbpy)_3_^2+^	Tris (4,4′-dicarboxylicacid-2,2′-bipyridyl) ruthenium(II) dichloride
91.	TiO_2_	Titanium dioxide
92.	NSs	nanosilks
93.	MoS_2_	Molybdenum disulfide
94.	QDs	quantum dots
95.	TiO_2_NSs@MoS_2_ QDs	Titanium dioxide (TiO_2_) nanosilks (NSs)@ Molybdenum disulfide (MoS_2_) quantum dots (QDs)
96.	HOTTIP	Homo sapiens HOXA distal transcript antisense RNA
97.	HepG2	immortal cell line
98.	LNCaP cells	Androgen-sensitive human prostate adenocarcinoma cells
99.	mDNAs	messenger DNAs
100.	Ru(NH_3_)_6_^3+^	Ruthenium trichloride
101.	miR-21	MicroRNA 21
102.	AAP	activatable aptamer probes
103.	CTCs	circulating tumor cells
104.	Ppy NWs	Polypyrrole nanowires
105.	GSH	glutathione
